# Heritability of language laterality assessed by functional transcranial Doppler ultrasound: a twin study

**DOI:** 10.12688/wellcomeopenres.15524.3

**Published:** 2020-09-16

**Authors:** Dorothy V.M. Bishop, Timothy C. Bates

**Affiliations:** 1 Department of Experimental Psychology, University of Oxford, Oxford, Oxon, OX2 6GG, UK; 2 Psychology Department, University of Edinburgh, Edinburgh, EH8 9JZ, UK

**Keywords:** laterality, language, genetics, twins, handedness, language lateralisation, functional transcranial Doppler ultrasound

## Abstract

**Background:** Prior studies have estimated heritability of around 0.25 for the trait of handedness, with studies of structural brain asymmetry giving estimates in a similar or lower range. Little is known about heritability of functional language lateralization. This report describes heritability estimates using functional language laterality and handedness phenotypes in a twin sample previously reported by Wilson and Bishop (2018).

**Methods:** The total sample consisted of 194 twin pairs (49% monozygotic) aged from 6 to 11 years. A language laterality index was obtained for 141 twin pairs, who completed a protocol where relative blood flow through left and right middle cerebral arteries was measured using functional transcranial Doppler ultrasound (fTCD) while the child described animation sequences. Handedness data was available from the Edinburgh Handedness Inventory (EHI) and Quantification of Hand Preference (QHP) for all 194 pairs. Heritability was assessed using conventional structural equation modeling, assuming no effect of shared environment (AE model).

**Results:** For the two handedness measures, heritability estimates (95% CI) were consistent with prior research: .25 (.03 - .34) and .18 (0 – .31) respectively for the EHI and QHP. For the language laterality index, however, the twin-cotwin correlations were close to zero for both MZ and DZ twins, and the heritability estimate was zero (0 - .15).

**Conclusions:** A single study cannot rule out a genetic effect on language lateralisation. It is possible that the low twin-cotwin correlations were affected by noisy data: although the split-half reliability of the fTCD-based laterality index was high (0.85), we did not have information on test-retest reliability in children, which is likely to be lower. We cannot reject the hypothesis that there is low but nonzero heritability for this trait, but our data suggest that individual variation in language lateralisation is predominantly due to stochastic variation in neurodevelopment.

## Introduction

Lateralisation of language and motor function in humans display two notable features: First, there is a pronounced population bias - to the left hemisphere for lateralisation of language, and to the right hand (controlled by the left hemisphere) for handedness; second there is individual variation in the direction and extent of lateralisation; a minority of individuals show a reversal of the typical pattern, and others show little or no asymmetry. The frequency of these atypical patterns will depend on how they are measured and defined: Vingerhoets (2019) estimated 6.5% of people have right language dominance and 10–15% have bilateral representation of language.

Other primates show some indications of lateralised functions, but humans are distinctive in their strong population bias to right-handedness. It is often assumed that evolution of manual laterality is related to development of a complex and lateralised language faculty in humans, but the biological origins of the population bias are not well understood for either trait. There is an association between handedness and language lateralisation, evident from a range of methods, including the impact of focal lesions, presurgical testing for language dominance in epilepsy, and imaging methods in healthy populations, but it is complex: whereas around 83–88% of right-handers have left-hemisphere language, this is true for around 64–68% of non-right-handers (see Carey & Johnstone, 2014, for a comprehensive meta-analysis including a range of methodologies for assessing language laterality).

Twins provide a useful natural experiment for estimating the contribution of genetic variation to individual differences in a trait. The twin method compares the similarity of identical (monozygotic or MZ) twins versus non-identical (dizygotic or DZ) twins to derive estimates of the relative contributions of genetic variants, environment shared by the twins, and other twin-specific influences (including chance) on individual variation in a trait. This is best understood intuitively by imagining a variety of hypothetical situations. In the first, the trait is solely determined by random chance: in that case, two members of a twin pair (A and B) would be no more similar than two unrelated people, and in a sample of twins, the correlation between twin A and twin B would be zero, regardless of zygosity. In a second fictitious scenario, the trait is solely determined by an environmental factor common to both twins, such as home environment. In that case, there would be perfect correlation between the traits for twin A and twin B, regardless of zygosity. In the third scenario, the trait is determined solely by genes: because MZ twins are genetically identical, the correlation between two members of a twin pair will be 1, but for DZ twins, who on average have 50% of their segregating genes in common, the correlation will be 0.5.

In practice, our goal is not to attribute variation in the observed trait (phenotype) to one cause or the other, but rather to estimate their relative contributions. The usual approach to twin analysis is to specify that the total variance in a trait, v is equal to a ^2^ + c ^2^ + e ^2^, where a is additive genetic variance, c is shared (common) environment and e is random, nonshared environment. Similarity between pairs of MZ twins is the sum of genetic and shared environmental influences, a ^2^ + c ^2^, and similarity between pairs of DZ twins is 0.5 a ^2^ + c ^2^, so we can estimate heritability (a ^2^) as twice the difference in correlation between MZ and DZ twins, and then obtain the values for c ^2^ and e ^2^ by simple algebra ( Sham, 1998). In contemporary twin research, this logic is implemented in a model-fitting approach, which makes it possible to test assumptions of the model and obtain standard errors of path estimates ( Eaves *et al.*, 1978).

Twin studies of manual laterality have shown that individual differences in handedness are largely determined by chance, with genetic variation playing a relatively minor role. A meta-analysis of 35 twin studies estimated heritability of around 0.23, with shared environment effect of zero ( Medland *et al.*, 2006).

Studies of language laterality are far less numerous, because of the difficulty and expense of studying large numbers of twins. Geschwind *et al.* (2002) wrote a paper entitled ‘Heritability of lobar brain volumes in twins supports genetic models of cerebral laterality and handedness’, but close inspection of the results reveals that the authors did not provide any evidence of heritability of structural brain asymmetry. Participants were 72 MZ and 67 DZ male twin pairs aged around 72 years. Conventional twin analysis was conducted and showed high heritability for volumes of the four lobes of the brain on the left and right. 86% of the MZ pairs and 88% of the DZ pairs were concordant for handedness. Further analyses were conducted to compare lobar volumes and asymmetries in those with consistent vs inconsistent handedness, but key data on MZ and DZ concordance for brain asymmetry were not presented. The general finding of high heritability for regional brain volumes is consistent with subsequent twin studies, but to throw light on genetics of cerebral asymmetry, we need data on MZ and DZ twin concordances for a laterality index that indicates the relative size of the two hemispheres. Such data were provided by Eyler *et al.* (2014), who studied 130 MZ and 92 DZ adult twin pairs and concluded: “Our findings suggest that genetic factors do not play a significant role in determining individual variation in the degree of regional cortical size asymmetries measured with MRI, although they may do so for volume of some subcortical structures.” (p. 1110).

Jahanshad *et al.* (2010) studied structural brain connectivity in 60 MZ and 45 same-sex DZ right-handed twin pairs using diffusion tensor imaging. They stated: “We expected genetic factors to play a substantial role in the lateralization of the fiber anisotropy in language association regions of the temporal lobe, including the arcuate fasciculus”, but in practice heritability estimates were modest at best. They started by looking at twin concordance in a voxel-wise analysis before moving to look at laterality in 12 regions of interest, concluding that genetic factors accounted for 33% of the variance in asymmetry for the inferior fronto-occipital fasciculus (part of the ventral language pathway), 37% for the anterior thalamic radiation, and 20% for the forceps major and uncinate fasciculus. Exclusion of left-handers from the sample may have led to inflated estimates of heritability, because most left-handed twins have a right-handed cotwin, and this discordance could be reflected in discrepant structural asymmetry for members of a twin pair. Neither this study nor that by Eyler *et al.* (2014) found any effect of shared environment on laterality: most variance was explained by the E (non-shared environment) term, reflecting a lack of correlation between both MZ and DZ twins.

Two recent studies by the ENIGMA consortium looked at brain asymmetry for subcortical and cortical structures respectively ( Guadalupe *et al.,* 2017; Kong *et al.,* 2018). The first study included analyses of 1170 individuals from 71 genetically informative pedigrees. Subcortical asymmetries, though small in magnitude, were significantly heritable for four of the seven regions, with genetic factors accounting for between .15 and .27 of variation ( Guadalupe *et al.,* 2017). Cortical brain asymmetries were more substantial, and in an increased pedigree sample, significant heritability was found for six of 34 measures of cortical thickness and four measures of area, after Bonferroni correction. These analyses were repeated in another genetically informative sample that included 143 MZ pairs and 85 DZ pairs; significant heritability for one of the thickness measures (parahippocampal gyrus) was replicated in this new sample, as was one of the area measures (superior temporal), with heritability estimates ranging from .15 to .23.

It is unclear how these structural asymmetries relate to functional language laterality; there was no evidence of any relationship with handedness in very large meta-analyses done by Guadalupe *et al.* (2017) or Kong *et al.* (2018).

We are aware of only three previous studies that assessed functional language lateralisation in a genetically informative design. Bryden (1975) used two different measures in both parents and two siblings from 49 families. Although he found one statistically significant association between mother and child, he drew attention to inconsistent findings – not only were the correlations between siblings negative, but one of the highest correlations was between mother and father. As he wryly noted, "This correlation would suggest non-random mating for laterality, a characteristic that one would hardly expect to be of significance in selecting one's spouse. "(p. 206). He noted that the measures had only moderate reliability (split half of .61 and .66) but concluded: "one should at least consider seriously the hypothesis that speech lateralization is primarily determined by environmental factors" (p.209).

Ocklenburg *et al.* (2016) used a behavioural measure of language lateralisation, relative ear advantage on dichotic listening, to estimate heritability from parent-offspring relationships in 103 families. Correlations between offspring and mid-parent were close to zero for a laterality index based on free listening, but significant heritability estimates of 0.28 - 0.36 were obtained when participants were instructed to direct attention to one ear. This pattern of results is complicated to interpret, as it could reflect a heritable impact on the ability to direct attention. The authors concluded that the findings "implicate a major contribution of non-genetic influences to individual language lateralization." Somers *et al.* (2015) assessed cerebral lateralisation using functional transcranial Doppler ultrasound in a multi-generational pedigree sample from a single community that had been geographically isolated for generations and so had low genetic heterogeneity. A potential advantage of the study was the use of a pedigree-based method of analysis, which gives higher power than a method reliant just on twin pairs. The selected sample was enriched for left handedness (309 people from 37 families). The heritability of handedness was estimated from pedigree data as 0.24, and the heritability of atypical language lateralisation (coded as a binary variable) was 0.31. The authors noted that heritability may have been overestimated because of oversampling of families with several left-handed members; selecting only families with at least two left-handers per generation could artificially inflate within-family similarity for laterality. Nevertheless, the heritability of handedness was similar to that obtained from other samples without such ascertainment bias. In both the Ocklenburg *et al.* study and the Somers *et al.* study, heritability was estimated from family relationships, ignoring any effect of shared environmental influences. This seems a reasonable assumption, given that none of the twin studies of laterality reviewed above has found an effect of shared environment.

### The importance of phenotype definition

One challenge for researchers studying cerebral lateralisation is how to conceptualise the phenotype. For cerebral lateralisation, it is possible to obtain a quantitative index reflecting the extent to which activation is more left- than right-sided; in fMRI a laterality index is commonly computed, where 1 is fully left, 0 is equal, and -1 is fully right. For handedness, a similar index may be computed, based on number of right-handed items endorsed on an inventory, relative skill of the two hands, or extent to which preference is maintained across the midline. Depending on how the index is computed, the distribution of scores may be strongly skewed to one side, or even bimodal.

For handedness, the non-normal distribution of preference scores has led to genetic models that propose that handedness is a mixture distribution formed by combining two underlying genotypes: one with a bias to right-handedness, and one with no bias ( Annett, 1985; McManus, 1985). However, a failure to find reliable genetic association of handedness with common variants led to an alternative view, which is that atypical lateralisation, of either hand or brain, is caused by any one of a large number of rare genetic variants that add noise to neurodevelopment ( Armour *et al.,* 2014). According to this view, there are numerous inherited causal mutations which would be expected to differ from family to family. However, within families, these mutations would be the same for MZ twins and their cotwins, whereas they would be identical on average in 50% of DZ twin pairs. Thus, twin models should be sensitive to such a heritable “neurodevelopmental noise” trait.

The genetic model of laterality that one adopts will affect the optimal analysis. If there are heritable influences on the whole continuum of lateralisation, then the standard method of twin analysis may be the best approach, although an ordinal approach may be needed for non-normal data. If, however, there are distinct genetic influences leading to a skewed laterality distribution, where there is a mixture of two underlying phenotypes, then an ‘extremes analysis’ may be more appropriate ( Bishop, 2005). This approach, pioneered by DeFries & Fulker (1988), involves identifying extreme cases (probands) from a twin sample. Insofar as genetic factors are involved, it is expected that the scores of their cotwins will fall below the population mean, with this effect being stronger for MZ twins, who have all genetic variants in common, compared with DZ twins, who share only 50% of genetic variants on average.

### The current study

As far as we are aware, to date there has not been a twin study that uses a direct, functional measure of cerebral lateralisation for language. We report genetic analysis from a study of 141 twin pairs, showing that, consistent with previous studies of handedness and brain asymmetry, chance (or environmental factors not shared between twins) plays the major role in determining individual differences in language lateralisation.

Our sample consists of twin children recruited for a study of the genetic bases of developmental language disorder (DLD), for whom language lateralisation was assessed using functional transcranial Doppler ultrasound (fTCD). Data from these children have previously been reported in the context of an analysis focusing on relationships between cerebral lateralisation and language functioning ( Wilson & Bishop, 2018). That analysis found no difference in language laterality between children with language disorders and those with typical language development, despite the internal consistency of the laterality index obtained with this measure being good (split-half reliability for odd and even trials = 0.84). Furthermore, comparison with a previous study using the same methods confirmed that language lateralisation in twins did not differ from that observed in single-born children. This sample provides a useful opportunity to fill a gap in the literature with an analysis comparing MZ and DZ twins in order to estimate the relative contribution of genetic and non-genetic variation to individual differences in language laterality.

## Methods

### Participants

For a detailed account of selection of participants, see Wilson & Bishop (2018). In brief, we recruited 194 pairs of twins aged 6 years 0 months to 11 years 11 months, using a sampling approach with the aim of including around 75% twin pairs where one or both had parental report of language or literacy difficulties. Our previous analysis found no association between language laterality and language disorder, and so all children were treated together here. Using a broad definition of language problems, including any mention of history of speech-and-language therapy or communication difficulties, out of 96 MZ twin pairs, 41 (43%) were concordant for language problems, 24 (25%) were discordant for language problems, and the remaining 31 (32%) had neither twin with language problems. Of the 98 DZ twin pairs, 21 (21%) were concordant for language problems, 44 (45%) were discordant for language problems, and the remaining 33 (34%) had neither twin with language problems.

Handedness assessments were completed for all children, and language laterality assessment (see below) was available for 141 pairs. The breakdown of the sample by zygosity and gender is shown in Table 1.

**Table 1.** N twin pairs by zygosity and sex.

Twin type	All	With fTCD
MZ female	49	30
MZ male	47	35
DZ female	32	25
DZ male	29	21
DZ male/female	37	30
Total	194	141

MZ = monozygotic; DZ = dizygotic; fTCD = functional transcranial Doppler ultrasound.

### Zygosity determination

(The following paragraph is copied from Newbury *et al*., 2018). Oragene kits (OG-500, DNA Genotek Inc, Ontario Canada) were used to collect saliva for DNA analysis from children with SCTs and their parents and available twin pairs. DNA extraction was performed using an ethanol precipitation protocol as detailed in the standard protocol (DNA genotek). All extracted DNA was genotyped on the Infinium ‘Global Screening Array-24 (v1)’, which includes 692824 SNPs including rare and common variations. Data were processed in the Illumina BeadStudio/GenomeStudio software (v. 2.03) and all SNPs with a GenTrain (quality) score of < 0.5 were excluded at this stage. All genotypes were further filtered using PLINK software v1.07 ( Purcell *et al.*, 2007); as recommended by Anderson *et al.* (2010), samples with a genotype success rate below 95% or a heterozygosity rate ±2 SD from the mean were removed, as were SNPs with a Hardy-Weinberg equilibrium P < 0.000001 or a minor allele frequency of less than 1%. Identity data within families and twin-pairs were used to exclude samples with unexpected gender or relationships. SNPs that showed an inheritance error rate > 1% or skewed missing rates between genotype plates were also excluded.

DNA was available for 191 twin pairs who were compared across 250,875 SNPs. All gave unambiguous zygosity signals on Identity by State (IBS), i.e., the proportion of SNPs for which any given twin pair share genotypes: this was either close to 1.0 (MZ) or close to 0.5 (DZ). For twins with missing or inadequate DNA samples we relied on parental report of zygosity.

### Laterality assessment

1. Handedness

Hand preference was assessed using a hand preference battery based on items from the Edinburgh Handedness Inventory (EHI) ( Oldfield, 1971), modified to exclude one item deemed unsuitable for children (striking a match). With adults, the EHI is administered as a questionnaire, but in our study children were asked to demonstrate each of ten actions: writing, drawing, throwing a ball, using scissors, using a toothbrush, cutting with a knife, using a spoon, using a broom (upper hand), taking the lid off a box, and dealing cards. One point was awarded for exclusive right hand use, zero points for left hand use, and half a point if both hands were used, giving a score ranging from zero to ten.

Strength of hand preference was assessed using the Quantification of Hand Preference (QHP) task ( Bishop *et al*., 1996), which measures the tendency to continue to use the preferred hand when cards are picked up from different spatial locations. Three cards are set out in each of seven positions extending at 30 degree intervals from the left to the right of the child’s midline. The child is not told that handedness is being assessed, and treats the task as a picture-name matching game, where they have to pick up the named card and put it in a centrally-placed box. The same quasi-random order of positions was used for all children, starting with a card at the midline and continuing until the child had picked up and placed three cards at each of seven locations, to give a total of 21 trials. For each card, two points were awarded for right-handed use, zero points for left-handed use, and one point if the card was transferred from one hand to another in the course of placing it in the box. Test-retest reliability of the QHP in adults has been shown to be good when there are five items in each position ( Doyen & Carlier, 2002), but it should be noted that a more recent study with 6- to 7-year-old children using 3 items per position found test-retest reliability of only 0.35 ( Pritchard *et al*., 2019); results from this test should therefore be interpreted cautiously.

2. Language laterality

Language laterality was assessed using functional transcranial Doppler ultrasound (fTCD) recorded while the child described short episodes from a story presented as an animation. The equipment consisted of Doppler-Box ^TM^ X digital (Smart Medical) with QL software. A DiaMon® monitoring headset was used with two 2 MHz hand-held probes (2.9m length). A video demonstration of this procedure using an earlier version of the equipment and headset is available from Bishop *et al.* (2010). Transcranial Doppler ultrasound is used in medical contexts to assess the integrity of the cerebral blood vessels. For assessing cerebral lateralisation, left and right ultrasound probes are attached to a headset and positioned so as to detect lateralised changes in blood flow in the middle cerebral arteries.

On each trial, the child silently views a 12 s clip from a cartoon that included sounds but no speech. A response cue appears when the video clip finishes to indicate the start of a 10 s period during which the child is asked to describe what happened in the cartoon. A second cue then indicates that the child should stop talking and relax. This paradigm has previously been found to have good validity and internal consistency ( Bishop *et al.* 2009). In adults, we recently demonstrated test-retest reliability of 0.84 for a Sentence Generation task that was similar to the task used here, but with static pictures rather than video sequences as stimuli ( Woodhead *et al.*, 2019).

A maximum of 30 trials was administered, depending on the child’s tolerance of the procedure. The child’s verbal responses were recorded and subsequently transcribed, and the examiner noted behaviour during the procedure. Trials were excluded if the child either spoke during a silent period, or failed to talk during the ‘talk’ period: these need to be omitted because they invalidate the trial, which involves comparing the period when the child talks with a baseline period when no talking occurs.

The analysis of the animation task data consists of a standard sequence of processing steps, following original work by Deppe *et al.* (1997). We used a custom script written in R ( R core team, 2019) for data processing. This included an initial step of identifying trials where there was very brief signal dropout (affecting one datapoint) and interpolating the mean value in such cases. Trials with more prolonged signal dropout were discarded. After these preliminary steps, heart cycle integration was applied to remove the heartbeat, followed by signal normalisation, artefact rejection, epoching and baseline correction. The averaged left and right velocity plots were subtracted to give a difference waveform.

Following Wilson & Bishop (2018) we excluded data from 11 twin pairs where one of the twins had fewer than 12 accepted trials, as the LI is likely to be unreliable when based on such a small amount of data. In addition, data were excluded for one twin pair where one child’s laterality index was more than 5 SD from the mean.

In our previous report of data from this sample ( Wilson & Bishop, 2018), we used the conventional approach for obtaining a laterality index based on the peak difference (maximum or minimum) in a period of interest, predefined as 4 to 14 seconds after the cue to speak. This method involves finding the largest maximum or minimum in the difference wave, and measuring the size of the difference for a 2 s period around that peak. Our subsequent studies with adults suggested that this method is not ideal, because there are cases where the difference wave shifts from positive to negative, or vice versa, within the period of interest, and quite minor differences in size of positive and negative peaks can determine whether laterality is coded as left or right ( Woodhead *et al*., 2018). Accordingly, in more recent studies, we have calculated a laterality index (LI) as the mean amplitude of blood flow velocity difference in the whole period of interest (4 to 14 s). In the current dataset, the correlation between this mean-based LI and the traditional peak-based LI is very strong: Pearson correlation = 0.911, DF = 280, but the distributions differ. The split-half reliability, based on correlation of LIs from odd and even trials is closely similar to that from the original method, r = 0.85. The original method, however, gives a non-normal distribution of laterality indices, with a point of rarity around zero, which appears to be a spurious artefact of the method of computation. As requested by reviewers, results obtained with the original peak method are included in our analysis for completeness, and the two methods are compared in a scatterplot in Appendix 1 ( https://osf.io/tfyk3/).

The standard error of the LI for each individual was computed from the LI obtained across individual trials. This allowed us to consider whether the child’s LI was significantly different from zero, i.e. whether the 95% confidence interval spanned zero. Where this was the case, laterality was categorised as left or right, and where the LI was not significantly different from zero, the laterality was coded as bilateral. Note that coding of bilateral laterality can result if data are merely noisy. For comparison with Somers *et al.* (2015), we also categorised individuals on the basis of the peak-based laterality index into 'typical' and 'atypical' laterality, with the latter group including all those who were not significantly left-lateralised (i.e., the confidence interval of their laterality index spanned, or fell below, zero).

Following a suggestion by Francks (2019), we also conducted genetic analysis of the mean left- and right-sided blood flow measures.

## Procedure

Sections of this paragraph are copied from Wilson & Bishop, 2018. Ethical approval was obtained for the study in 2011 from the Berkshire NHS Research Ethics Committee (reference 11/SC/0096), and data collection started in August of that year, finishing in October 2016. Information sheets, consent forms and ethics approval documents are available on Open Science Framework. Where families had expressed interest in the study, they were interviewed by telephone to assess whether the children were likely to meet inclusion criteria, and an appointment was made to see the twins at home or at school, depending on parental preference. Families were widely dispersed around the UK, including Northern Ireland, Scotland, Wales and England, so testing was scheduled where possible to minimise travel. During the course of recruitment, which lasted for a period of five years, a total of eight research assistants as well as the senior author were involved in assessing children. In some cases, two testers worked together, each seeing one twin, and in others a single tester saw both children sequentially. The assessment was conducted in a single session lasting between 2–3 hours per child, with breaks where needed.

## Data analysis

1. AE modeling

As noted above, the usual approach to twin analysis involves decomposing variance of a phenotype into components attributed to additive genetic (a), common (shared) environment (c) and nonshared environment (e). This decomposition is typically implemented using structural equation modeling with maximum likelihood estimates ( Rijsdijk & Sham, 2002), which make it possible to test whether the data meet underlying assumptions, to compare fit of different models, and to obtain standard errors of estimates. Large samples are needed to accurately estimate additive genetic (a ^2^), shared environmental (c ^2^) influences, both of which lead to positive covariance between two members of both MZ and DZ pairs: they are distinguished by the fact that genetic influence leads to greater covariance for MZ than for DZ twins. In the context of laterality, however, prior studies have found shared environmental influences to be negligible in adequately powered twin studies, and it is safe to ignore the c ^2^ term ( Medland *et al*., 2006; Medland *et al*., 2009). This simplifies the analysis, making it possible to detect genetic effects with smaller samples, as any positive correlation between twins and their cotwins can be interpreted as a genetic effect. An AE model was fitted to the raw data using two R packages (version 3.6, R Core Team, 2019): OpenMx package (verson 2.13.2) ( Neale *et al.*, 2016) with the umx package (version 3.0.0) used for the non-normal handedness data ( Bates *et al.*, 2019). Both univariate and multivariate models were evaluated. Scripts used to pre-process Doppler files are provided as extended data ( Bishop, 2019).

2. Power analysis

**Figure 1.**
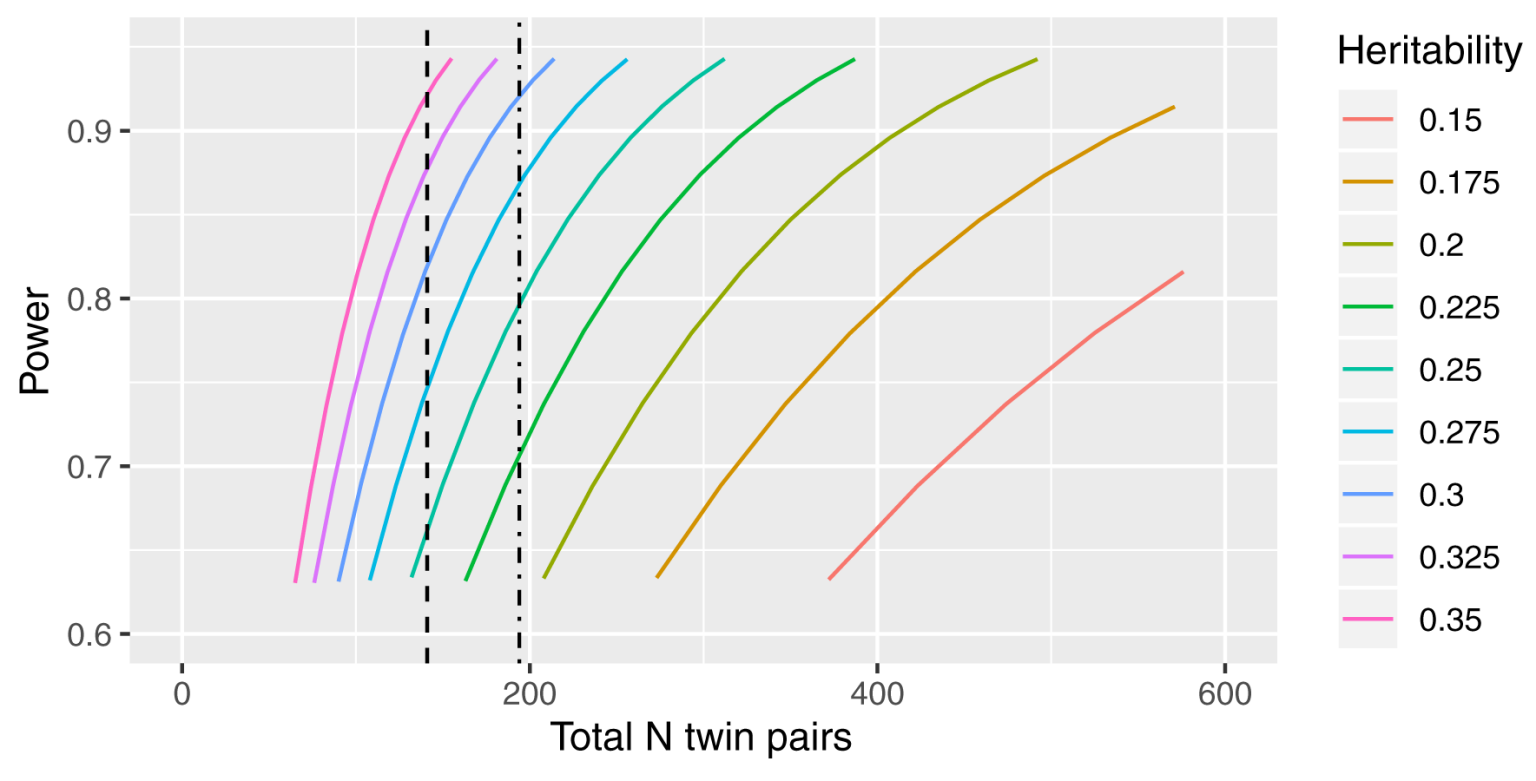
Power in relation to sample size for different levels of heritability in an AE model. The two vertical lines correspond to the sample size for handedness measures (right-most line) and the laterality index (left-most line).

A power analysis was conducted to estimate the power to detect heritability of 0.15 or more, using the power.ACE.test function in umx (see Figure 1). This showed that for the larger sample of twins for whom handedness data were available, there is 80% power to detect heritability of 0.25 in an AE model with alpha of 0.05, and for the smaller sample with language laterality data, power of 67%. In this smaller subset with language laterality data, 80% power is obtained for heritability of 0.3, and over 95% power for heritability of 0.35. Thus, even the low heritability handedness estimates reviewed in the Introduction should be detectable with this sample. For language laterality, there is around a 2 in 3 chance of detecting a true but small effect of around 0.25, and strong power to detect the higher heritability reported in the DTI study by Jahanshad *et al*. (2010). However, neither sample is adequately powered to detect heritability levels below .2

## Results

**Figure 2.**
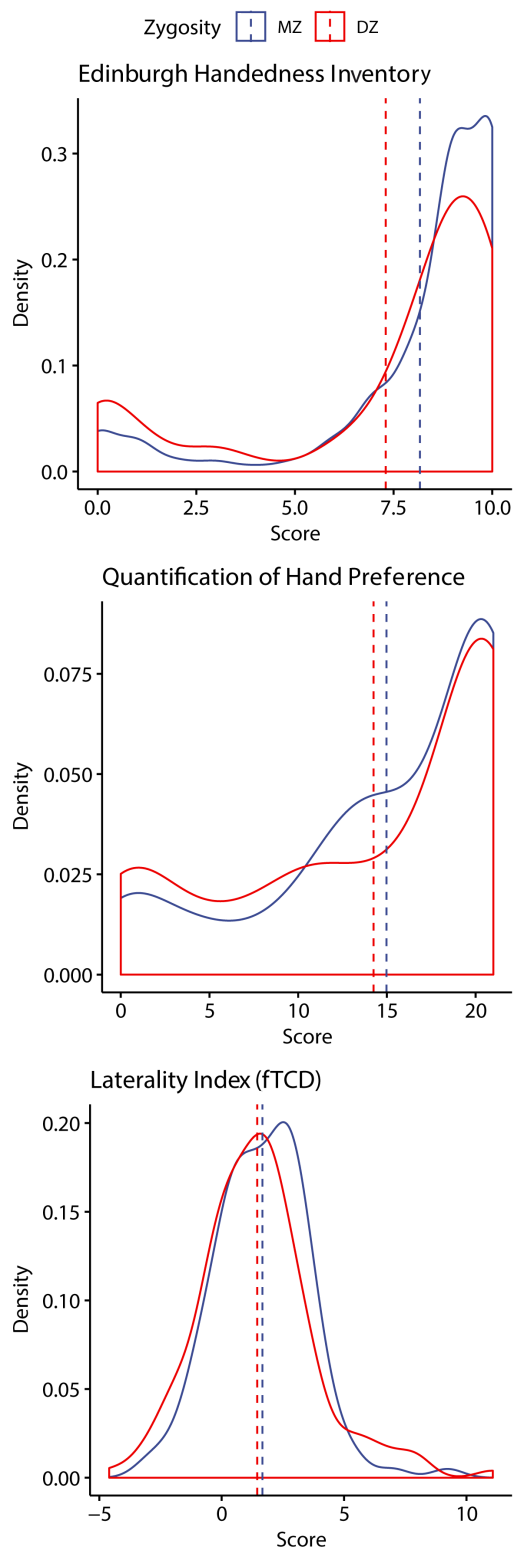
Vertical dotted line shows mean for MZ (blue) and DZ (red) twins. Density plots for handedness and language laterality indices.

**Figure 3.**
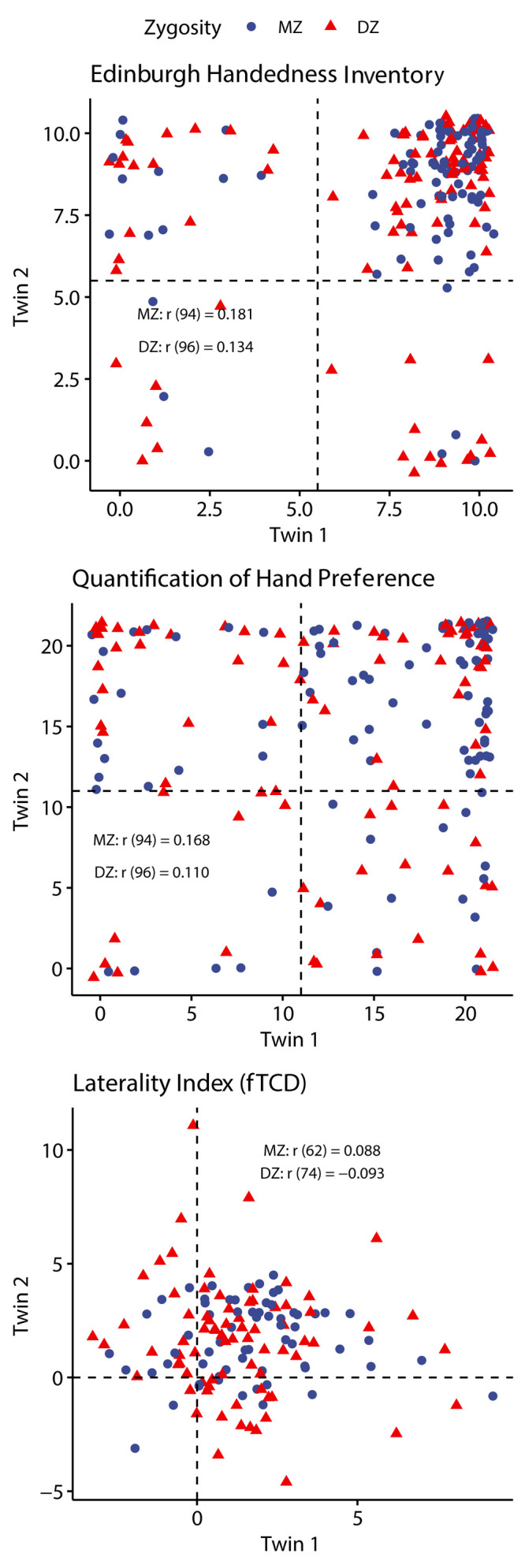
Scatterplots by zygosity for handedness and language laterality indices. The data from handedness measures are jittered; Spearman correlation coefficients are shown.

Figure 2 shows the distributions of scores obtained on the two handedness measures and the language laterality index from fTCD, and Figure 3 shows scatterplots depicting the association of the laterality indices between two members of a twin pair (see underlying data ( Bishop, 2019)).

The density plots reveal that data from the handedness measures are highly non-normal, following the usual J-shaped distribution for handedness measures, with the majority of cases bunched up at the right handed end of the scale.

Figure 3 shows that the correlations between two members of a twin pair are low for all measures.

Contrary to expectation, no association was found between handedness and the language laterality index: with cases divided into those with laterality indices above and below zero, 76% of the left-handers and 78% of the right-handers were left-lateralised for language.

The OpenMx package was used to run an AE model with the data from the two handedness tasks and the language laterality task. It was anticipated that the model would not fit with the data because (a) the handedness data were highly non-normal, and (b) the correlations for the language laterality index were close to zero. However, a good fit was obtained for all three measures. Heritability estimates for the two handedness measures were compatible with those obtained in previous studies, with values of a ^2^ of 0.25 for the Edinburgh Handedness Inventory and 0.18 for the Quantification of Hand Preference task (see Table 2). The fit of a model including a genetic term was substantially better than for one excluding it for both tasks (p-values less than 0.01 for both measures). For the language laterality index, the estimated value of a ^2^ was zero, and a model with no genetic term gave as good a fit as one including it. The same was true for both the laterality index based on the traditional peak method, and for the binary category of typical/atypical laterality that was comparable to that used by Somers *et al.* (2015).

**Table 2.** Heritability estimates from AE model for different measures. 95% CI in brackets. MZ = monozygotic; DZ = dizygotic.

Measure	MZ *	DZ *	rMZ	rDZ	a ^2^	chisq	p **
Edinburgh Handedness Inventory	96	98	0.18 (0.04 to 0.32)	0.13 (-0.01 to 0.28)	0.25 (0.03 to 0.34)	11.7	< .001
Quantification of Hand Preference	96	98	0.17 (0.01 to 0.3)	0.11 (-0.05 to 0.25)	0.18 (0 to 0.31)	6.8	.009
Laterality index (fTCD)	65	76	0.09 (-0.08 to 0.26)	-0.09 (-0.25 to 0.06)	0 (0 to 0.15)	0	1
Laterality index (peak FTCD)	65	76	0.11 (-0.07 to 0.27)	-0.07 (-0.23 to 0.09)	0.02 (0 to 0.20)	0	.823
Binary typical (1)/atypical (0)	65	76	0.02 (-0.16 to 0.19)	0.02 (-0.13 to 0.19)	0 (0 to 0.25)	0	1

* N pairs ** Significance of change of fit if a ^2^ term dropped

Because of concerns that the non-normality of the handedness data could distort heritability estimates, analyses of the EHI and QHP were repeated using the umx package ( Bates, 2018) to run an ordinal version of ACE analysis. This gave slightly different estimates of heritability than the standard analysis using the AE model, with values of a ^2^ of 0.24 and 0.22 for EHI and QHP respectively, and c ^2^ close to zero.

We also ran a multivariate model that included laterality indices from EHI, QHP and fTCD, to see how far heritable influences are shared between measures ( Table 3). This revealed that some of the genetic influence on QHP was shared with EHP, and that there was no shared genetic influence with the language laterality LI from fTCD.

**Table 3.** Multivariate AE model estimates, unsquared standardized path estimates with SEs.

		Shared by all 3 measures		Independent of EHP		Specific to LI	
		Estimate	SE	Estimate	SE	Estimate	SE
A	EHP	0.458*	0.098				
A	QHP	0.243	0.124	0.343*	0.093		
A	LI	0.123	0.113	-0.152	0.123	0.000	0.213
E	EHP	0.889*	0.051				
E	QHP	0.401*	0.068	0.814*	0.043		
E	LI	-0.134	0.066	0.039	0.064	0.971*	0.039

Note for table 3: Asterisks denote paths that are statistically significant at .05 level.

As suggested by Francks (2019), we also did a twin analysis of the left and right blood flow volumes. The pattern of twin-cotwin correlations for these measures was quite different from that seen for the difference scores, with modest but significant correlations between twins and cotwins, but no evidence of a zygosity-specific effect. For left-sided blood flow, the twin-cotwin correlation (95% CI) was 0.40 (0.24 to 0.55) for MZ twins and 0.33 (0.18 to 0.46) for DZ twins; for right-sided blood flow, the correlation was 0.24 (0.07 to 0.39) for MZ twins and 0.32 (0.16 to 0.45) for DZ twins. This suggests that shared environment, rather than genetic similarity, drives twin-cotwin similarity. Repeating the analysis with age and sex residualised did not affect results. A CE model was fitted to each measure, and gave as good a fit as an ACE model, with significant (p < .001) c ^2^ estimates of 0.34 for left-sided flow and 0.27 for right-sided flow. The left- and right-sided flow measures were closely linked, and intercorrelated with r = 0.87.

The basic logic of the DeFries & Fulker (1988) method for analysing heritability of extreme scores is that if we select probands with extreme scores, then the scores of cotwins should regress more to the population mean for DZ twins than for MZ twins. The plausibility of such a model can be readily tested by selecting twins with an extreme score and then using a t-test to compare co-twin scores for MZ and DZ twins. Results of this analysis are shown for all three phenotypes in Table 4, which shows no reliable difference between cotwins for MZ and DZ probands. These data must be interpreted with extreme caution because of the small sample sizes, but they do not lend any support to the idea that atypical laterality is caused by a qualitatively different genetic process than normal range variation, either for handedness or for language laterality.

**Table 4.** Extreme probands, t-tests comparing MZ/DZ co-twins. MZ = monozygotic; DZ = dizygotic; QHP = Quantification of Hand Preference, LI = laterality index.

	Edinburgh Handedness	QHP	Language LI
N MZ	21	40	22
N DZ	40	58	39
MZ proband mean (SD)	1.43 (1.66)	3.61 (3.86)	-1.06 (2.89)
DZ proband mean (SD)	1.15 (1.42)	3.89 (4.22)	-1.24 (3.29)
MZ co-twin mean (SD)	6.86 (3.48)	13.46 (7.14)	1.34 (2.49)
DZ co-twin mean (SD)	6.58 (3.63)	14.22 (7.19)	2.14 (2.57)
t	0.30	-0.52	-1.19
df	42.3	84.4	44.9
p	0.768	0.606	0.239

## Discussion

The twin analysis of handedness data from this study gave results that were consistent with those from previous meta-analyses, with around 20% of variance accounted for by genetic factors. The language laterality index, however, gave results that were unexpected in two respects. First twin-twin correlations were close to zero for both MZ and DZ twins, and appeared therefore to be determined entirely by chance; second, there was no difference in rates of left-sided laterality between left-handers (76%) and right-handers (78%), with the latter figure being lower than usually found using other indicators of cerebral lateralisation ( Carey & Johnstone, 2014).

This raises the question as to the validity of the laterality index obtained using functional transcranial Doppler ultrasound. If chance is the principal determinant of the LI, is this just because the measure is unreliable within individuals? We do not have test-retest data on children, but it seems unlikely poor reliability is the whole explanation, for three reasons. First, the split half reliability of the LI in this sample is around 0.85, which indicates reasonable consistency from trial to trial across the testing session. With adults, we have explicitly considered test-retest reliability of the LI obtained with fTCD, and found that, while it varies from task to task, it is generally good, with test-retest correlation of 0.84 for the task that is most similar to the animation description task ( Woodhead *et al*., 2019). Second, as shown in Figure 3, the children studied here showed a robust bias to the left hemisphere at the group level. Third, the current result is broadly consistent with the handful of studies that have looked at structural or functional brain lateralisation. Although these have revealed some heritable laterality indices, these are typically small in magnitude. It is also compatible with a recent study sequencing the genomes of 33 subjects with right-hemisphere language dominance and 34 typical left-dominant subjects and finding no associated mutations distinguishing the individuals with atypical language laterality ( Carrion-Castillo *et al*., 2019).

When one considers that the levels of blood flow to left and right hemisphere show no indication of genetic influence, it is not surprising that the laterality index, based on the difference between these measures, is not heritable. The evidence for a shared environment effect on the blood flow measures is unexpected; one possibility is that the intrauterine environment could be implicated in influencing development of cerebral vasculature.

A further question is why language laterality shows zero heritability, whereas handedness shows small but significant heritability. This difference in findings may prove to be an uninteresting artefact of the smaller sample size for the language laterality measure than for handedness, combined with perhaps lower reliability of the measure, leading to reduced power to detect a true effect. As the difference in heritability estimates between measures was not large, we cannot dismiss the possibility that the true level of heritability for language laterality is similar to that for handedness - slight but not totally absent.

One does need to be cautious about assuming lack of genetic effect on the basis of a small sample. In the past, the first author concluded that handedness was not heritable, on the basis of small-scale twin studies that found no evidence of genetic influence, but subsequent meta-analyses have shown consistent but low heritability. It has nevertheless been remarkably difficult to find any genetic variants consistently linked with variations in handedness, and many promising findings appear to have been false positives ( de Kovel & Francks, 2019). It subsequently became clear, however, that there is a genetic effect, but it is small and only clearly detectable in large samples. In a sample of over one million people, Cuellar-Partida *et al.* (2020) identified 41 common genetic variants that were associated with left-handedness, and 7 associated with ambidexterity, each with a very small effect. While very low levels of heritability pose methodological problems, the search for genetic variants continues, not so much with the goal of explaining large amounts of variance in handedness, but rather with the goal of illuminating the biological pathways involved in determining asymmetry. This can be feasible, provided there are suitable phenotypic measures available in very large samples ( de Kovel & Francks, 2019; Wiberg *et al.,* 2019)

It is possible that the same will prove to be the case for language lateralisation, especially given prior findings of significant heritability in structural measures of subcortical brain regions ( Eyler *et al.,* 2014; Guadalupe *et al.,* 2017), cortical area and thickness ( Kong *et al.,* 2018) and language-related fibre tracts ( Jahanshad *et al.,* 2010), plus the large family study of Somers *et al.* (2015) that used a binary phenotype, and the mixed findings on dichotic listening by Ocklenburg *et al.* (2016). A further point to note is that different language tasks show different degrees of lateralisation ( Woodhead *et al.,* 2019), and we simply do not know which may be the most heritable. As Ocklenburg *et al.* (2016) noted: "there seems to be a considerable phenotype-dependent variability regarding the heritability of language lateralization." (p. 37). A research priority should be the development of optimal methods for deriving a valid and reliable language laterality index from brain measures, as without these, progress will be limited.

The current small study is not sufficient to prove zero heritability for lateralised brain function. On the other hand, it is striking how difficult it has been to replicate previous studies of genetic associations with laterality, and the flexibility with which the phenotype can be defined does increase the likelihood that some findings may be type I errors ( Bishop, 1990).

Our data are compatible with a more radical model in which language laterality is the consequence of a general population left-sided brain bias for language which is genetic but not heritable, i.e., does not show any individual variation. If a genetic biasing factor applies to the whole population, without there being any variation, then heritability will be zero. The postulated population bias mechanism would have to be at least somewhat probabilistic, with some individuals showing atypical lateralisation just by chance. Such a model is consistent with the view of neurodevelopment proposed by Mitchell (2018). He noted that it is customary to interpret the ‘e’ term of an ACE model as reflecting some systematic environmental influence that is not shared by the two members of a twin pair, literally ‘non-shared environment’. He argues that this neglects the likely role of stochastic influences on neurodevelopment (and in many traits), and notes that evidence for such ‘developmental noise’ comes from the numerous instances where there is phenotypic variability despite genetic identity. This is seen not only in MZ twins with the same genetic sequence but different phenotypic outcomes, but also in the two sides of the face, which are seldom totally symmetric, despite having the same DNA.

It would be rash to draw strong conclusions from a single study with a null result, especially when the expected level of heritability is low. It is possible that with larger samples and different measures we will be able to confirm that language lateralisation is a heritable phenotype. These results, however, encourage us to at least consider the provocative possibility that language lateralisation may be a phenotype that breaks Turkheimer’s (2000) first law of behaviour genetics: ‘All human behavioural traits are heritable’.

## Data availability

### Underlying data

Open Science Framework: Double entry data. https://doi.org/10.17605/OSF.IO/5h82q ( Bishop, 2019)

This project contains the following underlying data:

- doubleentry_data_dictionary.xlsx (Excel spreadsheet with data dictionary for TwinLatOSF.csv)
- Twins_Doppler_processed_NewLI.xlsx (CSV file containing handedness and language laterality data)
- heritability lat writeup_forpaper.rmd (R markdown script to create this paper with figures and analyses).
- Appendix 1: Scatterplot showing relationship between two methods of deriving laterality index (peak vs mean measures).

### Extended data

Open Science Framework: Preprocessing for mean LIs - twins. https://doi.org/10.17605/OSF.IO/CPKHB ( Bishop, 2019)

This registered project contains the following extended data:

- R_doppler_v2_NEW_LI_2019_DB.R (R script for preprocessing of Doppler files)
- Individual trial LIs.csv (CSV file containing individual language laterality file data)
- Doppler_raw.zip (Zip file containing individual raw Doppler readings)

Data are available under the terms of the Creative Commons Zero “No rights reserved” data waiver (CC0 1.0 Public domain dedication).
