# Supplementary material for: Heritability of language laterality assessed by functional transcranial Doppler ultrasound: a twin study
Source: Wellcome Open Res. 2020 Sep 16;4:161. Originally published 2019 Oct 28. [Version 3] doi: 10.12688/wellcomeopenres.15524.3 (PMC7194484; doi:10.12688/wellcomeopenres.15524.3)
Supplement: Peer review history

# Peer review: Reviewer response for version 3

**DOI:** 10.21956/wellcomeopenres.17905.r40488

**Published:** 2020-09-18

**Authors:** Chris McManus

**Affiliations:** 1 Research Department for Medical Education, University College London, London, UK; 2 Research Department of Clinical, Educational and Health Psychology, University College London, London, UK

Thanks to the authors for those minor but useful revisions. I am now very happy to recommend indexing of this interesting, unusual and important study.

Is the work clearly and accurately presented and does it cite the current literature?

Partly

If applicable, is the statistical analysis and its interpretation appropriate?

Partly

Are all the source data underlying the results available to ensure full reproducibility?

Yes

Is the study design appropriate and is the work technically sound?

Partly

Are the conclusions drawn adequately supported by the results?

Partly

Are sufficient details of methods and analysis provided to allow replication by others?

Yes

Reviewer Expertise:

Neuropsychology of cerebral lateralisation

I confirm that I have read this submission and believe that I have an appropriate level of expertise to confirm that it is of an acceptable scientific standard.

# Peer review: Reviewer response for version 2

**DOI:** 10.21956/wellcomeopenres.17276.r38147

**Published:** 2020-05-11

**Authors:** Chris McManus

**Affiliations:** 1 Research Department for Medical Education, University College London, London, UK; 2 Research Department of Clinical, Educational and Health Psychology, University College London, London, UK

Firstly my thanks to the authors for their helpful re-analyses and thoughtful comments on the revision. It was good to see that estimates I made of the upper confidence interval for the heritability were broadly correct, even if done by a different method. My apologies also for the delay in getting back – the revision arrived on my desk just at the same time as a vast amount of work arising in medical schools from the Covid-19 outbreak, and I have only just found a sufficient gap to be able to return to this very interesting and undoubtedly important study.

 Inevitably any revision, particularly with new data analyses, results in further questions being raised, and it is inevitable that some of those will require some sort of reply.

 The replies to the current revision by the author can broadly be divided into two groups. Those to do with the details of the data and the analyses, and those to do more generally with philosophy of science, and issues arising from the replication crisis. I will deal with the latter first.

**Philosophical issues**.  As the authors say, many of the criticisms, “get to the nub of issues that have become prominent in discussions of the so-called 'reproducibility crisis'”. Type II errors have always been problematic, particularly with low sample sizes (and this it has to be said is a very low sample size compared with many evaluating heritability).

*Acceptable studies in the previous literature*.  Discursive reviews had many problems, one of which was to review lots of studies, reject most as being inadequate in some form or another, and then conclude there were but a few decent bits of evidence, all of which happened to support one’s own theory. Meta-analysis in large part got rid of that, particularly if study quality measures were included, and the file drawer methods, funnel plots, etc, helped to deal with the problems of the literature being biased. Now however a new issue arises, as “in the absence of pre-registered studies, we also need to adopt a cautious stance to the prior literature”.  There are of course no other pre-registered studies in the literature except this one, which does a good job of cleaning the Augean stables, but sadly leaves almost no babies in their bath water (to mix metaphors). It does make life easier, of course, but medicine went through this a decade or two ago when some decided that the only treatments that should be used were those supported by randomised controlled trials. That fell apart when it was realised that there were no RCTs for penicillin, that there were no RCTs for treating common conditions such as ear wax, and that a decent RCT carried out for patients aged 50-69 could not be used if one happened to be aged 49 or 70…  Easy but strict rules make stupid decisions, and in general judgement is required. At this point I am tempted to say that there are also no pre-registered studies testing out what Newton or Darwin talked about…  You get the idea.

 P-hacking is another issue, but the solution is not to reject any choice of outcome measure, but instead perhaps to try all possible ways of dividing up outcome measures – variants of this in data science go under the heading “targeted learning” – and very complicated they can be!

 Enough said, but I personally am not going to throw away the entire previous literature as yet, and in particular I will tend to stick with what Broca and his successors found, rather than saying that they can safely be ignored. I will also continue to believe that it is at least plausible that the underlying genetic basis for lateralisations is held in common.

*Use of language as a philosophical issue*.  This is also a philosophical/methodological issue concerning the extent to which language biases the interpretation of otherwise relatively objective numbers. Thus to say in the discussion that, “the possibility that the true level of heritability for language laterality is similar to that for handedness – slight but not totally absent.”  “Slight” here is mainly being used to dismiss a result, rather than to reflect the reality of the presence of an association.  Whether the heritability of handedness is indeed “slight” or “small” (as earlier in the paragraph) depends on the interpretation of a heritability of 0.25 in the very large meta-analysis of Medland. Yes, 0.75 of the variance indicates what in the case of language, was called, “a major contribution of non-genetic influences to individual language lateralization” (Oklenburg *et al.*), but that something else is not necessarily environmental factors.  There is an elision here from not genetic to implying that it must therefore be environmental (and hence important). Measurement error is also a part of it, but also is what can be called ‘deep chance’ – quantum variation, or the chance processes by which random processes flip to right or left. They are part of the environment in some strict sense but not in any real sense. [Do we usually say that when a coin comes down heads or tails that it is due to ‘environmental variation’ – and if so, casino owners might get very worried]. My guess is that the majority of variation in handedness is due to randomness (and hence the C in the DC model), with only perhaps 5 or 10% at most of the variation due to real environmental variation. A full partitioning of the heritability would show that more clearly. Non-genetic is not environmental.

 The last line of the paper is nice – I always like researchers at least to consider “provocative possibili[ies]”, and hence that Turkheimer may have been wrong --but I will be putting my money on this one eventually being shown to be yet another example of the First Law being correct.  But that use of “slight” was also mainly provocative I suspect…

**The data and the analyses**. Lots of improvements here.

*Appendix 1* is useful, with its plot of peakLI versus mean LI in fTCD.  So useful that useful that I couldn’t help feeling that that it needs to be properly in the main paper if you strongly want to argue that fTCD and Peak fTCD are different. In fact they don’t look very different in Appendix 1. There is a notch, but the interesting thing is the individuals whose direction would be mis-classified in the two systems. There are 16 individuals whose meanLI is positive but whose peakLI is negative, which is perhaps not surprising. More unexpected is there being only 2 individuals who move from negative to positive. Imagine that the true distribution is normal with a mean of 2.5 and SD of 2.5, with the cut point at zero, so that about 16% have an LI less than zero. Imagine that only those within .5 units (0.2 SDs) of the cut point will be affected by the peak algorithm, and half of those above the cut point move below, and vice-versa for those below the cut point. If N=200 then overall about 32 have negative LIs (16%). Of those likely to move, 9 are below the cut point and 11 above the cut point, with half of each group moving. Overall therefore about 5% move, with the same proportion in each direction.  So why in the data do 16 go one way and only 2 the other?  I have my prejudices but I can help wondering whether there is actually a bimodal distribution underlying this?  All of that means that I think you should put appendix 1 into the main text and let people see it in clear sight. Electronic journals have few space limitations, and the point being made is an important one, particularly if you wish to claim that meanLI is better than peakLI.

*Table 2* is particularly useful with its inclusions of confidence intervals. It is worth setting these out more clearly:

 EHI                      .03 to 0.34

 QHP                    0 to 0.31

 LImean                 0 to 0.15

 LIpeak                  0 to 0.20

 Binary                   0 to 0.25

 Somers                 0.08 to 0.51

 [Somers is a rough estimate from the values that they give. You are welcome to get a better estimate, but I think the point will still be made.]

 Looking at these I find it very difficult to assert that we can say there is heritability for handedness (although of course the Summers meta-analysis makes that very clear) but that there is not heritability for language lateralisation, as the estimates clearly all overlap substantially,  or for that matter that the Somers estimate is in any major way different from the present one.

 Ultimately I fear that the main conclusion is that, large though the present study is in terms of studies of the inheritance of language lateralisation, neither this nor the Somers study is compelling evidence that there is or is not heritability for language lateralisation. Taking it one step further, there is also no compelling evidence from these data that the heritability of handedness is substantially different from the heritability of language. Of course we do know that the Summers meta-analysis says something very different for handedness (but Summers was not pre-registered so perhaps it should be thrown out as well [forgive!]). I’m sorry, but that is surely an honest assessment of what there is here. Bring in some Bayesian priors then it could be swung in other directions, but the heritability estimates are pretty clear as giving no solid answers either way.

*The abstract*. The previous paragraph makes it clear that these confidence intervals are important and interesting. They should therefore be in the abstract, as that is what most people will read of this study. So put them in there.

*The multivariate analyses*. I think the second author was right here, and that it should have been included, for to do so is to treat the readers as adults who can cope with complexity. Overkill might have been a good description if all the hypotheses were already dead and stiff, but they clearly are not. Looking at the table for the multivariate analysis, there is immediately a clear demonstration of EHI and QHP showing a co-inheritance of about 0.6 (and I suspect exactly the same inheritance as the upper limit seems to include 1).  I can’t say that it surprises me that EHI and QHP are mainly measuring the same thing, so that their covariation is mainly genetic, but it is of interest to those of us in laterality (and reassures us that actually the boring old EHI, whatever its problems, is probably doing as well as the elegant and much more theoretically justifiable QHP).

 The core of the multivariate analyses though is what LI is doing. Yes, the estimate is zero, but once again with an SE of .213, the upper limit seems to be about 0.4, which is robust, and throws . Whether there is co-inheritance of language and handedness is a key question (and one or two genetic  models suggest there may be), and the EHI/LI covariance of .123 (with its SE of .113) might suggest that, although it is difficult to be sure. Either way, I am sure that these numbers are better in the public domain, as a part of open science, rather than as a throw-away remark to me lurking in a reply to comments. I think the adults would would appreciate that.

*A minor point*. The authors might like to reconsider their comments in the discussion about the de Kovel and Francks 2019 study.  In particular, the more recent study by Gabriel Cuellar Partida, Medland, *et al*, in BioRxiv, http://dx.doi.org/10.1101/831321 ^1^, found 41 genetic loci related to handedness, with a narrow sense heritability of 12%.  There is also a reassuring similarity to the 2013 ANYAS paper by McManus, Davison and Armour which, “estimate[d] at least 40 loci are involved in determining handedness” doi: 10.1111/nyas.12102 ^2^.

**Summary**

 This review has been rather discursive, and is partly a review of a response to a review, so let me say what I think is needed for this paper to be acceptable:

- The abstract should contain standard errors and confidence intervals.
- The paper should contain the table summarising the multivariate analyses.
- The paper should contain the figure from Appendix 1 showing the relationship between the conventional LI peak measure and the new LI mean measure.
- A reference to Cuellar Partida and Medland should probably be included.
- The authors don’t necessarily need to change much else, but they may wish to. Personally I would have included a figure showing all of the heritabilities, with their confidence intervals, which would make clear the uncertainties surrounding all of the estimates.

Is the work clearly and accurately presented and does it cite the current literature?

Partly

If applicable, is the statistical analysis and its interpretation appropriate?

Partly

Are all the source data underlying the results available to ensure full reproducibility?

Yes

Is the study design appropriate and is the work technically sound?

Partly

Are the conclusions drawn adequately supported by the results?

Partly

Are sufficient details of methods and analysis provided to allow replication by others?

Yes

Reviewer Expertise:

Neuropsychology of cerebral lateralisation

I confirm that I have read this submission and believe that I have an appropriate level of expertise to confirm that it is of an acceptable scientific standard, however I have significant reservations, as outlined above.

## References

[1] : Genome-wide association study identifies 48 common genetic variants associated with handedness. *bioRxiv*.2019; 10.1101/831321 10.1101/831321 PMC711662332989287

[2] : Multilocus genetic models of handedness closely resemble single-locus models in explaining family data and are compatible with genome-wide association studies. *Ann N Y Acad Sci*.2013;1288: 10.1111/nyas.12102 48-58 10.1111/nyas.12102 23631511PMC4298034

# Response

**Published:** 2020-09-01

**Authors:** Dorothy Bishop

**Affiliations:** University of Oxford, UK

We thank Chris McManus for his thorough reading and evaluation of the paper which has helped us improve the paper further.

Re the point about non-genetic not being the same as environmental, we are in total agreement. This is discussed in the final section, where Mitchell's emphasis on stochastic processes is alluded to.  The last sentence of the abstract makes the same point. McManus, and Annett, in their genetic models, were ahead of the field in attributing a substantial part of variance in heritability to chance.

**Specific recommendations for revision**

Re the specific recommendations in the report by McManus: we have attended to these, with the following exceptions:

We have not incorporated the scatterplot comparing mean and peak methods, which is in an Appendix. We agree that the best way to measure LI is far from certain, and there is a realistic possibility that there is underlying bimodality. Adopting a mean measure seems the optimal approach in the face of uncertainty, because if you take a continuous measure when the underlying reality is bimodal, then nothing is lost: indeed the comments by McManus about movement in each direction suggest one way to explore whether a continuous distribution fails to fit expectations. But if you assume bimodality when the underlying reality is continuous, the peak method induces a spurious bimodality, by creating an artefactual dip in the distribution close to zero. The reason for omitting the plot in the main paper is because  (a) this paper is about heritability, rather than measurement questions, and to digress into a discussion of that issue would distract from the main point, and (b) it really doesn't make any difference to the kinds of quantitative analysis we report here – the difference between measurement methods becomes much more important if the focus is on categorical/quantitative aspects of laterality. The data are available to those interested in this issue, but are a distraction in the context of this paper.

We have not added a further figure to show the heritability estimates with CIs, as Table 2 provides this information.

# Peer review: Reviewer response for version 2

**DOI:** 10.21956/wellcomeopenres.17276.r38149

**Published:** 2020-04-14

**Authors:** Guy Vingerhoets

**Affiliations:** 1 Ghent University, Ghent, Belgium

The authors have responded clearly to all my comments. I’m happy to approve the manuscript, pending correction of some minor typos in the power analysis section (percentages). In the same section I also find the sentence “For language laterality, there is around *a 23 in 34 chance* of detecting a true but small effect of around 0.25,…” somewhat difficult to grasp. Perhaps better expressed as a percentage?

Is the work clearly and accurately presented and does it cite the current literature?

Yes

If applicable, is the statistical analysis and its interpretation appropriate?

Yes

Are all the source data underlying the results available to ensure full reproducibility?

Yes

Is the study design appropriate and is the work technically sound?

Yes

Are the conclusions drawn adequately supported by the results?

Partly

Are sufficient details of methods and analysis provided to allow replication by others?

Partly

Reviewer Expertise:

Neuropsychology.

I confirm that I have read this submission and believe that I have an appropriate level of expertise to confirm that it is of an acceptable scientific standard.

# Peer review: Reviewer response for version 2

**DOI:** 10.21956/wellcomeopenres.17276.r38148

**Published:** 2020-03-17

**Authors:** Clyde Francks

**Affiliations:** 1 Language and Genetics Department, Max Planck Institute for Psycholinguistics, Nijmegen, The Netherlands; 2 Donders Institute for Brain, Cognition and Behaviour, Radboud University, Nijmegen, The Netherlands

Many thanks to the authors for responding positively and thoughtfully to my earlier comments. I have only some minor points at this stage:

1. In the results section there are confidence intervals reported for the MZ and DZ correlation coefficients, for the separate analyses of L and R blood flows. However, for the main analysis of the laterality index, I think these correlations are only shown in Figure 3, without confidence intervals. The correlations and their confidence intervals for the laterality index could be included in the text. The finding of a shared environmental component to L and R blood flows suggests that a CE model could be tested for the laterality index, although the MZ and DZ correlations for the laterality index already indicate that C will not be significant.
2. There are typos in the section on power analysis (805%, 6773%). The heritabilities in the power section have three or four figures after the decimal point.
3. Please see the Discussion section of this paper about handedness, which also addressed randomness in early development (in the context of low heritability and weak associations with early life factors): de Kovel *et al*. (2019) ^1^.

Is the work clearly and accurately presented and does it cite the current literature?

Partly

If applicable, is the statistical analysis and its interpretation appropriate?

Partly

Are all the source data underlying the results available to ensure full reproducibility?

Yes

Is the study design appropriate and is the work technically sound?

Yes

Are the conclusions drawn adequately supported by the results?

Partly

Are sufficient details of methods and analysis provided to allow replication by others?

Yes

Reviewer Expertise:

Human neurogenetics.

I confirm that I have read this submission and believe that I have an appropriate level of expertise to confirm that it is of an acceptable scientific standard.

## References

[1] : A large-scale population study of early life factors influencing left-handedness. *Scientific Reports*.2019;9(1) : 10.1038/s41598-018-37423-8 10.1038/s41598-018-37423-8 30679750PMC6345846

# Peer review: Reviewer response for version 1

**DOI:** 10.21956/wellcomeopenres.16993.r36875

**Published:** 2019-11-26

**Authors:** Chris McManus

**Affiliations:** 1 Research Department for Medical Education, University College London, London, UK; 2 Research Department of Clinical, Educational and Health Psychology, University College London, London, UK

This is a complicated and difficult paper to review, not least because the central finding, of zero heritability for language dominance, is unexpected given both theory and other studies in the literature which have looked at the inheritance of cerebral asymmetries, including language and handedness, and their inter-relation. It has to be said immediately that the opening word of the title, “Negligible”, is perhaps unnecessarily provocative given the potential problems of the present study.

*Expectations*. The abstract begins by saying that “it is widely assumed that individual differences in language lateralisation have a strong genetic basis”. [Without going further into the issue, referring to effects as ‘strong’ or ‘small’ or ‘low at best”, are terms that not well defined, and suggest more of rhetoric than scientific argument; and I note in passing that I do it myself in this review]. Few studies of the inheritance of lateralisation argue for strong effects since the deep randomness of fluctuating asymmetry tends to preclude strong effects. The use of “assumed” suggests a lack of substance to any genetic basis for lateralisation, but that is surely not the case. Handedness is undoubtedly under genetic control in part, and most data suggests that handedness and language lateralisation are correlated (although again that is often described as ‘small’, although the tetrachoric correlations are of the order of 0.7). Various genetic models also suggest that handedness and language dominance may well be under control of the same genetic systems. Even if the single gene models of Annett and McManus are wrong, the multilocus model of McManus *et al.* (Ann NY Acad Sci, 2013) ^1^, the companion piece to the cited Armour *et al.* paper ^2^, fits well to twin and family data. Although the calculations are not in the paper, the same logic as for the single gene DC model also explains the expected genetic link between handedness and language dominance. The present study also describes the Ocklenburg *et al.* heritability for language dominance of 0.28-0.36 ^3^ , and the Somers *et al.* heritability of 0.31 ^4^. All of that is more than “a wide assumption” but a strong *a priori* in Bayesian terms. Is it therefore the case that the present zero heritability for language lateralisation is a rare case that breaks Turkheimer’s first law of behaviour genetics ^5^? It is a strong claim, and strong evidence is needed, with it being clear that there are not other methodological issues which make it inconsistent with the rest of the literature. 

 The present study with its reasonably large number of twin pairs, may be unique in the published literature, to my knowledge. However although language lateralisation is difficult and expensive to measure using fMRI, Badzakowa-Trakjov *et al* (2010; not referenced in the current paper) ^6^ included data on 34 MZ pairs and 11 DZ pairs (with data being available from the authors); they did not however calculate heritabilities. The Human Connectome Project with its 132 MZ and 101 DZ pairs, for which data are available for download, has information on handedness and probably has measures related to language dominance (although I haven’t dug into that vast set of information), and again heritabilities will be calculable. Finally, the rapidly-growing UK Biobank has large amounts of data including brain scanning on about 18,000 individuals at present, which should rise to 100,000 soon; and twin pairs have been identified, so that eventually there could be perhaps 300 MZ and 600 MZ pairs. Some mention is perhaps worth making of other data sources.

*Heritabilities. *A weakness of the present study is the absence of information on confidence intervals of estimates, particularly of heritability.

 A starting point is the heritability of handedness, which is graphed in Figures 2a and 2b, with heritabilities in Table 2, of .190 for EHI and .170 for QHP. These are consistent with existing data and seem to provide reassurance that there is sufficient power in the present study for identifying heritability. Not being provided with confidence intervals, I looked at the raw data in more detail. Considering just EHI, the raw correlations from Figure 2 are .181 and .134 for MZ and DZ twins. Bootstrapping the data (R=10,000) to get a confidence interval (stratified analysis in R using boot() ) gives 2.5 ^th^ and 97.5 ^th^ percentiles for the correlations of MZ and DZ twins of -.049 to .426 and -.081 to .339, which are wide and of the order expected given the Ns (96MZ and 98 DZ pairs). Calculating heritability (a ^2^) from the bootstrapped data with umxACE() gives a 95% confidence interval of zero to 0.506, with a median of 0.232, consistent with the estimate in the paper. Overall, 21.5% of bootstrapped heritability estimates were effectively zero (<0.0001). None of that is unexpected given the overall sample size for twins, and the fact that heritability of handedness in twins is only really robust across very large samples in meta-analyses. It does however raise difficult questions for the robustness of the estimate of heritability in language dominance in the present study, with its somewhat smaller Ns.

 The key data for estimating heritability of fTCD LI in the present study are the MZ and DZ correlations in Figure 2 which are .088 and -0.093. The negative DZ correlation immediately suggests that any standard modern calculation of heritability is likely to be exceedingly low. It should also be noted that Ns are smaller than for handedness, with 65MZ and 76 DZ pairs. The bootstrapped MZ correlation has a 95% range of -.096 to 0.487 (median=.176) , and the DZ correlation has a 95% range of -.184 to .264 (median = .024). Given that 12% of MZ correlations and 42% of DZ correlations are less than zero, it is unsurprising that 85% of heritability estimates were 0, although some were positive, the 97.5 ^th^ percentile being of 0.117, and the 99.5 ^th^ and 99.95 ^th^ percentiles being 0.195 and 0.310. The 95% confidence interval for the fTCD is therefore about 0 to 0.117. Whether that includes “negligible” is debatable.

 In summary, although the number of twin pairs is large in conventional terms, the statistical power of the study is relatively low, as can be seen by the 95% confidence interval for the handedness data from 0 to .506 (in a possible range of 0 to 1). For language dominance the confidence interval is from 0 to 0.11, with most estimates being zero, which is a result of many of the correlations being negative, or DZ correlations being greater than MZ, and again that reflects the sample size (heroic though it may be in practical terms).

*More sophisticated genetic models*. Genetic models of handedness and language dominance generally assume – and are effective in so doing – that a single genetic system causes both handedness and language dominance, albeit they are not correlated perfectly due to random variation (which is indistinguishable from error variance but is actually due to deep chance). It would seem sensible therefore to fit a bivariate genetic model to the handedness and language dominance data. I tried it on the raw data, and there was little evidence of shared genetic variance between the handedness and language dominance phenotypes, but that is hardly surprising given the zero heritability for language dominance. Bootstrapping is more complex, and I haven’t tried it but it should be done. 

*Language dominance and handedness*. Most work on language lateralisation has found that atypical lateralisation is correlated with left-handedness (and for instance the Badzakowa-Trakjov *et al.* paper found a correlation of .357 (p<.001) between word generation and handedness ^6^). Many other studies find a similar correlation (and the present paper quotes Vingerhoets (2019) ^7^ with estimates of 6.5% of right handers and 10-15% of left-handers having atypical language laterality, although the latter estimate may be on the low side). The uncited meta-analysis by Carey and Johnstone (2014) ^8^ contains a systematic review using different methods of assessing language dominance and finds risk ratios of 1.36 for Wada studies, 1.22 for dichotic/tachistoscopic studies, 1.21 for fMRI/TDS/ECT studies. Considering just the eight fTCD studies, of 516 right-handers, 6.4% showed atypical dominance, compared with 41.2% of 369 left-handers.

 The question immediately arises as to the correlation of language dominance and handedness in the present study, which is not, I think, reported. The correlation between fTCD LI and the EHI is -.0642, p=.2841. Using writing hand and fTCD LI dichotomised around zero as Left or Right, 21.4% of 238 right-handers and 23.8% of left-handers have atypical language lateralisation which is clearly not significant. Also of interest is that 21.7% of all participants seem to have atypical language lateralisation which is higher than is usual and raises questions about the measure of language lateralisation and the sample.

*Calculating the laterality index for fTCD*. Language lateralisation indices in most previous studies using fTCD have followed Deppe *et al.* and assessed the maximum difference between right and left flow ^9^. That might well have problems since values of zero are inevitably made very unlikely, and results in a dip around zero. The present study uses a new algorithm based on calculating the mean difference between flow in the right and left arteries during the event window. That seems sensible, but it is not at all clear whether the results are equivalent. A previous study (Woodhead, Rutherford and Bishop, 2018) ^10^ compared the Deppe method with the mean method and reported a correlation of laterality indices of 0.97, although data were not shown and the participants were almost entirely right-handed. Whether means were different was unclear, and a scattergram would have been useful. An advantage is claimed to be that the “bimodality of the laterality index distribution is not seen when the means-based method is used”, although that is not self-evidently good when there are strong *a priori* expectations that laterality indices may well be bimodal. The present paper doesn’t include I think the Deppe method indices in the data files and therefore no further exploration could be carried out.

 Taken overall, the lack of an association of language lateralisation with handedness – which was found in most other previous studies – coupled with a high proportion of atypical language lateralisation overall, and a new method of data calculation does raise worries that the present results are in part artefactual. It would be reassuring to know that precisely the same results were obtained when the data were processed with DopOSCCI.

*The sample. *Most studies using fTCD have used undergraduate participants or typically developing children. Although it is not mentioned in the abstract, the present participants are from the Wilson and Bishop (2018) ^11^ study where the study, “us[ed] a sampling approach with the aim of including around 75% twin pairs where one or both had parental report of language or literacy difficulties”. The present paper reports that 43% of MZ pairs were concordant for language difficulties compared with 21% of DZ pairs (p.5). The implication is not only that many of the sample have language or literacy problems, with 55% of MZ twins and 44% of DZ twins having problems, but that also it is probable that there is an inherited component. Fitting an AE model gives estimates of heritability of .279 (CI = .130 to .416). Overall it seems clear that this population is probably far from representative of the general population. The results should probably be interpreted carefully.

*Summary*. Interesting though this study is, there are multiple reasons to treat it with great care, and in particular the headline conclusion of “negligible heritability of language laterality” may be somewhat overstated. There are potential problems with:

- the relatively small sample size for a twin study;
- the wide confidence intervals for heritability of handedness which generally does show heritability in twins but here has a lower confidence interval of zero;
- a 95% bootstrapped confidence interval for the heritability of language lateralisation which has an upper limit of 0.11;
- the lack of bivariate modelling of handedness and language dominance with a single underlying gene;
- the lack of an association of handedness and language lateralisation, although most studies find such an association, for which there are theoretical expectations;
- the use of a new algorithm for calculating the laterality index which also appears to give a high rate of atypical dominance, and the lack of calculations using the traditional algorithm;
- the sample not being representative of the population but being selected for a high rate of language and literacy difficulties which themselves appear to be heritable.

Is the work clearly and accurately presented and does it cite the current literature?

Partly

If applicable, is the statistical analysis and its interpretation appropriate?

Partly

Are all the source data underlying the results available to ensure full reproducibility?

Yes

Is the study design appropriate and is the work technically sound?

Partly

Are the conclusions drawn adequately supported by the results?

Partly

Are sufficient details of methods and analysis provided to allow replication by others?

Yes

Reviewer Expertise:

Neuropsychology of cerebral lateralisation

I confirm that I have read this submission and believe that I have an appropriate level of expertise to confirm that it is of an acceptable scientific standard, however I have significant reservations, as outlined above.

## References

[1] : Multilocus genetic models of handedness closely resemble single-locus models in explaining family data and are compatible with genome-wide association studies. *Ann N Y Acad Sci*.2013;1288: 10.1111/nyas.12102 48-58 10.1111/nyas.12102 23631511PMC4298034

[2] : Genome-wide association study of handedness excludes simple genetic models. *Heredity (Edinb)*.2014;112(3) : 10.1038/hdy.2013.93 221-5 10.1038/hdy.2013.93 24065183PMC3931166

[3] : Investigating heritability of laterality and cognitive control in speech perception. *Brain Cogn*.109: 10.1016/j.bandc.2016.09.003 34-39 10.1016/j.bandc.2016.09.003 27643948

[4] : Linkage analysis in a Dutch population isolate shows no major gene for left-handedness or atypical language lateralization. *J Neurosci*.2015;35(23) : 10.1523/JNEUROSCI.3287-14.2015 8730-6 10.1523/JNEUROSCI.3287-14.2015 26063907PMC4589567

[5] : Three Laws of Behavior Genetics and What They Mean. *Current Directions in Psychological Science*.2000;9(5) : 10.1111/1467-8721.00084 160-164 10.1111/1467-8721.00084

[6] : Cerebral asymmetries: complementary and independent processes. *PLoS One*.2010;5(3) : 10.1371/journal.pone.0009682 e9682 10.1371/journal.pone.0009682 20300635PMC2837380

[7] : Phenotypes in hemispheric functional segregation? Perspectives and challenges. *Phys Life Rev*.2019; 10.1016/j.plrev.2019.06.002 10.1016/j.plrev.2019.06.002 31230893

[8] : Quantifying cerebral asymmetries for language in dextrals and adextrals with random-effects meta analysis. *Front Psychol*.2014;5: 10.3389/fpsyg.2014.01128 1128 10.3389/fpsyg.2014.01128 25408673PMC4219560

[9] : AVERAGE: a Windows® program for automated analysis of event related cerebral blood flow. *Journal of Neuroscience Methods*.1997;75(2) : 10.1016/S0165-0270(97)00067-8 147-154 10.1016/S0165-0270(97)00067-8 9288646

[10] : Measurement of language laterality using functional transcranial Doppler ultrasound: a comparison of different tasks. *Wellcome Open Res*.2018;3: 10.12688/wellcomeopenres.14720.2 104 10.12688/wellcomeopenres.14720.2 30345386PMC6171558

[11] : Resounding failure to replicate links between developmental language disorder and cerebral lateralisation. *PeerJ*.2018;6: 10.7717/peerj.4217 e4217 10.7717/peerj.4217 29333343PMC5764032

# Response

**Published:** 2020-02-22

**Authors:** Dorothy Bishop

**Affiliations:** University of Oxford, UK

**Response to reviewers**

Our thanks to all three reviewers for their thoughtful comments on this article. There was strong concordance in their critiques, and so we will respond to the general points that were made before going on to cover specific points by individual reviewers.

**General comments**

This paper, and the criticisms, get to the nub of issues that have become prominent in discussions of the so-called 'reproducibility crisis'.  The fundamental question is how do we determine whether a null result, like the one reported here, is a type II error. We agree with the reviewers that it is not enough just to present a null result and conclude that the true effect is zero. A null result could arise because the study is underpowered to detect a true but small effect, or because the methods lack reliability or validity. In the Discussion we considered these possibilities and concluded that we could not draw a strong conclusion about our null finding, though we could specify a likely upper bound for heritability.  However, the use of the word 'negligible' in the title attracted criticism from all reviewers, and we accept that this is too value-laden and have deleted it.

Another point is that when evaluating research findings we should never rely on evidence from a single study. Again, we noted in the Discussion how Bishop's earlier findings of insignificant heritability of handedness had been overturned by subsequent, larger studies, which emphasises the need for caution. The suggestions by reviewers to try other analyses to see if the results look different are consistent with a Bayesian approach that demands especially strong evidence to overturn a strong prior belief. 

There is, however, a need for caution here.  Prior expectations come from at least two sources. First, there is the issue of needing a plausible mechanism, and we agree that a genetic basis for individual differences in a neurobiological phenotype is plausible. The main source of priors, however, will be previous literature. All three reviewers have cited additional sources for genetic influences on laterality. But there is a question of just how much confidence one should place in prior literature, given that there are many inconsistent findings, plus three systematic biases that distort results: publication bias, p-hacking and citation bias.  It is always difficult to discuss these biases, because it looks as if one is singling out other researchers for criticism, and impugning their integrity. Nevertheless, their prevalence is not in doubt (Greenwald, 1975; Sterling, Rosenbaum, & Weinkam, 1995; Fanelli, 2012), and there is circumstantial evidence that they have influenced the field of laterality.  Consider, for instance two twin studies, one on handedness by Davis and Annett (1994), and one on structural brain asymmetry by Geschwind et al (2002). Both studies are interpreted by their authors as supporting genetic models of cerebral lateralisation, but neither reported heritability estimates for laterality (or the zygosity-based correlations that underpin these), despite having the data available for doing this. One conclusion is that the heritability estimates were not convincing, and so went unreported.

The role of publication bias in distorting beliefs about solidness of findings was nicely documented in a simulation by Nissen et al (2016). Where this effect is compounded by p-hacking, then we can end up with up with solid beliefs based purely on the fact that we are sampling biased evidence. On top of that we all have a tendency to confirmation bias, which means that even when null findings are published, we tend to disregard them, which further biases the evidence (Bishop, 2020).

The field of laterality research is at particular risk of bias because there are so many different ways of conceptualising the phenotype. This point was made with regard to handedness by Bishop (1990), who noted that if you do not prespecify in advance how you plan to convert a handedness scale into groups, then you raise the chance of finding a 'significant' result to well above 5%.  This is equally true for other types of laterality, where there is no agreement about accepted measurement practices. And where laterality measures are part of a larger battery of tests, then it is likely that results on heritability will usually be published only if significant. The reviewers note the need for cautious interpretation of our results, and we agree, but in the absence of pre-registered studies, we also need to adopt a cautious stance to the prior literature, as there is a substantial risk of type I error. Large sample sizes can save us from type II errors but they are no defence against type I errors if p-hacking is possible. Findings that have been replicated using the same methods can be given much more weight than one-off studies.

Interpretation of evidence in this field is further complicated by the fact that there are many different forms of laterality – as well as handedness we have both structural and functional brain laterality.  Once we move from handedness, little is known about the reliability of these different measures of phenotype, but it is clear that they are not interchangeable, and the relationships between them are not clearly understood.

These points are amplified below when dealing with specific points raised by reviewers; the final paragraph of the Discussion has been amended to make it clear that we are not claiming that we have definitely proven a null result, but rather that very low, or even absent heritability of functional language lateralisation should at least be treated as a realistic contender, rather than dismissed as implausible.

**Responses to specific comments by reviewers**

**1. Clyde Francks**

1.1. *Request for confidence interval for heritability estimate and goodness of fit statistics*

These have now been provided in Table 2.

1.2. *Additional papers*

Thanks for drawing our attention to these papers that include genetic analysis of structural brain asymmetries. We should not have overlooked these papers which are indeed familiar to the first author, but in mitigation neither paper mention the words genetic or heritable in the title, and only Guadalupe et al mentioned 'heritability' in the keywords, so it is easy to miss these when conducting a systematic search for relevant papers. They are now included in the account of structural asymmetries.

The recent GWA studies of handedness are now mentioned in the Discussion, and we note that while these show very low SNP-based heritability, this does not preclude successful gene mapping – though the sample sizes required make it unlikely this will be feasible using  measures of language function based on fMRI or fTCD. Unfortunately, UK Biobank did not include language function activation methods in fMRI.

1.3 *Alternative pipeline for Doppler method*

*CF notes: "Somers et al. (2015) ^5^ found a heritability of 31% for atypical language lateralisation (coded as a binary variable), using functional transcranial Doppler ultrasound in a multi-generational pedigree sample. If the same data processing pipeline from that study would be applied in the current study, might the results no longer be discrepant between the two studies?*"

Somers used the traditional peak-based approach to analysing the fTCD data. Although for some analyses they used a continuous laterality index, heritability was reported only for a binary category of typical vs atypical. Correspondence with Dr Somers confirmed that this method was not suited to continuous data.

Results from analysis of peaks is now added to Table 2 for completeness, but please note that one reason for abandoning the peak approach is that it forces the data into a bimodal distribution. We moved away from this approach when we found that there were some individuals with L-R difference waves that hovered around zero: depending on whether the peak difference was greater for L or R, the peak would be taken at that point, and for those with very slight differences between the sides, this could seem fairly arbitrary. See Woodhead, Rutherford and Bishop, 2018, for discussion of this point.

1.4 *Heritability of L and R*

*CF notes: “In general, measuring asymmetry necessarily involves calculating some kind of difference score, which means that error variance in both left and right measures can affect the asymmetry measure. This may partly explain the low heritabilities of asymmetry indexes, and the authors could acknowledge this. In brain structural analysis (of e.g. grey matter volumes, surface areas, thicknesses), we typically see higher heritabilities for L and R separately than for the asymmetry index (L-R)/(L+R). Might it be informative to calculate heritabilities for L and R, in the context of functional transcranial Doppler sonography? If L and R are not themselves heritable, there may be a problem with the approach.”*

This is an interesting suggestion, which we have adopted. The correlation between the L and R flow measures *within* individuals is very high (close to .9). As now described in the paper, the raw L and R mean flow measures showed significant twin-twin correlations, but there was no effect of zygosity, suggesting that the similarity between twins was partly due to shared environment, rather than genetic influences. This is an intriguing result, but it is hard to interpret. This is not an age effect: age was not correlated with the flow measures, and residualising scores on age and sex did not make any difference. There have in the past been environmental explanations proposed for handedness, including shared in utero environment, which we have now alluded to. CF implies that a lack of heritability of the individual L and R flow measures may indicate a problem with the approach, but another possibility is that this trait is simply not under genetic control. We are not aware of any previous literature on heritability of cerebral blood flow.

1.5. *Support for assumption of strong heritability*

*Abstract: The authors state that ‘it is widely assumed that individual differences in language lateralisation have a strong genetic basis’. Are there references to support this? I am not sure that many researchers working on the genetics of laterality have this impression (this one does not).*

This comment has been removed, as it is based solely on informal impressions – mainly surprised reactions when these results are discussed.

1.6 *Rewording*

*The Discussion can be revised in parts, to reflect the recent literature indicated above.*

*The authors wrote that ‘Our data are compatible with a more radical model in which language laterality is the consequence of a general population left-sided brain bias for language which does not show any individual variation.’ For clarity, it would help to make explicit that genetic variation is meant here. I agree that the author’s data are compatible with this model, but the limited sample size means that the data are also compatible with a heritability up to whatever level is encompassed by the confidence interval around the best estimate.*

We have reworded as requested

2. **Guy Vingerhoets**

2.1 *Methods clarification*

*The only detail that puzzles me is the period of interest of the fTCD procedure. In one paragraph a start and stop cue indicating a 10s period is mentioned during which the child describes the 12s cartoon. In another paragraph the period of interest is defined as 4 to 14s after the cue to speak, but this method was abandoned for the ‘whole period of interest’. Would that be the 10s period between both cues then?*

This has now been written more clearly to explain how the peak method works, and how it differs from the mean method.

2.2 *Validity of fTCD laterality*

*fTCD- derived LI’s offer only crude estimations of asymmetry as they will pick up velocity changes due to co-activation of many other mental components that take place in the MCA territory and that may not be related to language. The child has just seen a 12s cartoon and now must describe it. Attention, memory, visual imagery, movement(?), receptive and productive language areas all come into play and most of these functions will be associated with activation of the lateral cortex. Besides not being very region-specific, fTCD does not allow for the use of control-tasks that can correct for task-unspecific activation.*

This is a point that is often raised by those who work with fMRI, and the Oxford group has given it much consideration – for instance, using tasks based on Mazoyer et al's sentence generation and control tasks, Woodhead et al (2018) computed laterality indices based on the difference in lateralised activation for sentence vs list generation.  We showed this made little overall difference, because list generation was not lateralised. The central point is that while, of course, there are numerous nonlinguistic factors involved in performing the animation description task, any nonlateralised activity is subtracted out by our analytic procedure, when we take the difference score. One can see in the waveforms associated with different tasks periods when blood flow increases and decreases – e.g. the blood flow increases just before starting to talk, then falls away. But so long as these are symmetrical effects they do not affect the laterality index, which is based on the difference waveform.

2.3 *Relative size of effect for fTCD and fMRI*

We are currently doing some direct comparisons of laterality indices from fMRI and fTCD with adults, and are finding good levels of agreement of laterality indices for equivalent tasks, but we would caution against attempting direct comparisons of magnitude of effects, because of the very different ways in which brain activation is measured.  With fMRI a general linear model is used to measure how the brain activation relates to experimental design variables, and then t-statistics of the voxels within the left and right hemisphere ROIs are computed and thresholded, and either the count (extent) or the sum (magnitude) of supra-threshold t-values is calculated in each hemisphere. With fTCD, the blood flow signal is epoched, normalised and baseline corrected, so that both left and right sensors have an average signal of zero in a resting time period at the start of each trial. The intensities of the left and right signals are then directly compared within a period of interest when the participant is performing the task.

**3. Chris McManus**

3.1 *Plausibility of laterality based on prior literature*

*CM "The use of “assumed” suggests a lack of substance to any genetic basis for lateralisation, but that is surely not the case."*

All reviewers challenged this statement, so it has been removed. We would stress that we are not arguing that all laterality is non-heritable, and we agree that as regards handedness, there is enough evidence to be confident in heritability of around .25, and growing evidence of genetic influences on structural brain asymmetry in some regions (as now reviewed more fully).

However, this is not the same as functional language laterality, where there is a paucity of evidence. As far as we are aware, the sum total of prior evidence is contained in 3 studies:

a) Ocklenburg, Bryden, and Somers. CM mentions the Ocklenburg study that we cited. This found zero heritability for the standard dichotic listening task, but heritability of .28 to .36 for a task condition with directed attention. The authors of that study concluded that their results: "implicate a major contribution of non-genetic influences to individual language lateralization."

b) Bryden (now mentioned in our revision), used two measures in both parents and two siblings from 49 families.  Although he found one statistically significant association between mother and child, he drew attention to inconsistent findings – not only were the correlations between siblings negative, but one of the highest correlations was between mother and father. As he wryly noted, "This correlation would suggest non-random mating for laterality, a characteristic that one would hardly expect to be of significance in selecting one's spouse. " (p. 206).  He concluded: " ..the present study has failed to find any particularly compelling evidence for a genetic basis for speech lateralization. While the problems associated with the use of an indirect measure of only moderate reliability may have doomed this study from the start, it does suggest that one should at least consider seriously the hypothesis that speech lateralization is primarily determined by environmental factors" (p.209). The split half reliability of the measures was .61 and .66.

c) The strongest evidence for heritability of language laterality on a functional brain measure is from the Somers et al study mentioned above using fTCD with a multi-generational pedigree sample from an isolated community. The heritability of atypical language lateralisation (coded as a binary variable) was 0.31.  This sample was not at all representative of the general population: it was deliberately selected to have relatively low genetic heterogeneity and to include families with several left-handed members. This might affect generalisability of findings, but the most serious limitation of the sample was that the selection method was biased toward phenotypic similarity of those who were in the sample; i.e. insofar as handedness is related to language laterality, then selecting only families with at least two left-handers per generation could artificially inflate within-family similarity for laterality.  On the other hand, a potential advantage of the study was the use of a pedigree-based method of analysis, which gives higher power than a method reliant just on twin pairs.

Taken together, the evidence is far from compelling, with moderate reliabilities, modest samples sizes, and heritability estimates compatible both with zero and with modest values such as .3 or so.

3.2 *Other studies*

*CM notes "Badzakowa-Trakjov et al (2010; not referenced in the current paper) ^6^ included data on 34 MZ pairs and 11 DZ pairs (with data being available from the authors); they did not however calculate heritabilities."*

This was not cited precisely because it is hard to derive any information about heritability from the study, because the sample was not only small, but also was selected to be biased to include discordant pairs (half the pairs studied had discordant handedness). Following the prompt from CM, we downloaded the data (available from PLOS One). The correlation between 34 MZ twin pairs on Word Generation was -.11 and for 11 DZ pairs it was .32. This is hardly encouraging for a genetic theory of language lateralisation, but, for the reasons noted above, it would be rash to draw much of a conclusion from this.

*The Human Connectome Project with its 132 MZ and 101 DZ pairs, for which data are available for download, has information on handedness and probably has measures related to language dominance.*

As CF points out above, there are some analyses of structural asymmetries. As far as we know there are no data on functional asymmetry

Biobank: again, there are analyses of handedness, but, although there are MRI data on a subset of individuals, the functional MRI in Biobank did not include a language task. We believe there are moves afoot to derive a laterality measure from resting state fMRI, but it is unclear how this would relate to laterality on something like a word or sentence generation task.

3.3 *Need for confidence intervals*

Thanks for the computations of confidence intervals around heritability estimates – these were helpful and in line with our bootstrapped computations, which are now given.

3.4 *Multivariate models*

The second author in fact had suggested we include these, but the first author thought this would be overkill, given that, as the reviewer points out, it would involve testing whether zero heritability in one trait is shared with low heritability in another. These results are given here for completeness, but not incorporated in the main paper. Path estimates are shown for the AE model, as all C estimates were zero.

Unsquared standardized estimates of paths from multivariate model.

Asterisks denote paths that are statistically significant at .05 level.

  Term Measure Estimate.EQL SE.EQL Estimate.QL SE.QL Estimate.L  SE.L

1    A     EHI        0.458*            0.098        

2    A     QHP      0.243             0.124       0.343*           0.093        

3    A     LI           0.123             0.113      -0.152            0.123      0.000    0.213

4    E     EHI        0.889*            0.051        

5    E     QHP      0.401*            0.068       0.814*          0.043       

6    E     LI         -0.134              0.066       0.039           0.064       0.971    0.039

N.B. EQL loadings for paths to all three measures: QL paths independent of EHI; L paths specific to language LI.

Information Criteria:

      |  df Penalty  |  Parameters Penalty  |  Sample-Size Adjusted

AIC:      5562.0751               8922.075                 8923.893

BIC:      -489.8496               8976.597                 8929.033

3.5 *Language dominance and handedness*

Relevant data on this are  now added.

We note also that the lack of association is inconsistent with previous literature which tends to find a significant association between handedness and laterality measures.

3.6 *Numbers with atypical dominance*

*CM notes "A further query about validity of the data is raised by the relatively high proportion with atypical lateralisation."*

With fTCD, the proportions with atypical lateralisation are entirely dependent on the language measure used, as shown by Woodhead et al (2018). This leads us to conclude that language lateralisation is not a fixed binary property of the brain, but varies in degree according to task demands. Some discussion of this point is now added.

3.7 *Use of the Deppe original method*

*Whether means were different was unclear, and a scattergram would have been useful. An advantage is claimed to be that the “bimodality of the laterality index distribution is not seen when the means-based method is used”, although that is not self-evidently good when there are strong a priori expectations that laterality indices may well be bimodal.*

A scattergram has now been added in the Appendix. We disagree regarding the 'strong a priori expectations': there are some theories that treat handedness as bimodal, but others that do not, and, for language laterality, there is no particular reason to assume bimodality.

Data on LIs obtained using the 'peak' method are now added.

*CM states "It would be reassuring to know that precisely the same results were obtained when the data were processed with DopOSCCI."*

Please see Woodhead et al (2018) where we note: " the R script had been developed in our group to fulfil the need for a reproducible and efficient method for processing large numbers of datasets, without using commercial (Matlab) software that required a licence (see Wilson & Bishop, 2018). As with DopOSCCI the analytic pipeline closely followed procedures developed by Deppe et al. (2004), with one additional option: the possibility of identifying brief periods of signal spiking or dropout and interpolating over these, to avoid rejecting trials. Wilson and Bishop (2018) compared results from  DopOSCCI and the R script and found only small differences in the LIs computed by the two methods". Given that we have spent a great deal of time developing scripts that allow us to process the data efficiently in R, and checking that it gives results highly consistent with the prior DopOSCCI approach, we do not think it reasonable to be asked to revert to the prior method.

Everyone makes errors of course, and we cannot rule out that there may be a bug somewhere, but we feel it is unreasonable to expect us to keep analysing our data using different methods. We do agree that there is an element of arbitrariness in the data processing pipeline for fTCD – results will vary depending on the sequence of operations, treatment of outliers, the period of interest, and whether peaks or means are used, but in our experience these differences have only minor impact on the final laterality index. We are interested to evaluate other approaches, but the method presented here was judged to be optimal and is the best we can do at present.  Our scripts are available, together with the raw data, so others are welcome to try different approaches.

3.8 *Sample*

*Overall it seems clear that this population is probably far from representative of the general population.*

While this is true, in our prior paper, we showed that there was no difference in cerebral lateralisation for children with and without language problems, and similar results to those obtained by Groen et al (2012) with singleborn children.  Prior studies have found no evidence for any genetic link between language disorders and lateralisation (Bishop, 2001; 2005).

**Overall**

The criticisms offered by the reviewers are generally fair, and we are happy to moderate the way in which the current results are described to clarify the limited conclusions that can be drawn. For the reasons stated at the outset, it is important that null results are published so that future meta-analyses are not biased in favour of positive findings. We hope that there will be more studies on this topic so that in future it might be possible to incorporate these data in a meta-analysis, to give a more precise estimate of heritability of language laterality.

**References**

Bishop, D. V. M. (1990). How to increase your chances of obtaining a significant association between handedness and disorder. *Journal of Clinical and Experimental Ñeuropsychology, 12*, 786-790.

Bishop, D. V. M. (2001). Individual differences in handedness and specific language impairment: evidence against a genetic link. *Behavior Genetics, 31*, 339-351.

Bishop, D. V. M. (2005). Handedness and specific language impairment: a study of 6-year-old twins. *Developmental Psychobiology, 46*, 362-369.

Bishop, D. V. M. (2020). The psychology of experimental psychologists: Overcoming cognitive constraints to improve research. The 45th Sir Frederic Bartlett Lecture *Quarterly Journal of Experimental Psychology, 73*(1), 1-19. doi:10.1177/1747021819886519

Bryden, M. P. (1975). Speech lateralization in families: A preliminary study using dichotic listening. *Brain and Language, 2*, 201-211.

Fanelli, D. (2012). Negative results are disappearing from most disciplines and countries. *Scientometrics, 90*(3), 891-904. doi:10.1007/s11192-011-0494-7

Greenwald, A. G. (1975). Consequences of prejudice against the null hypothesis. *Psychological Bulletin, 82*(1), 1-20.

Nissen, S. B., Magidson, T., Gross, K., & Bergstrom, C. T. (2016). Publication bias and the canonization of false facts. *eLife, 5*, e21451. doi:10.7554/eLife21451.001

Sterling, T. D., Rosenbaum, W. D., & Weinkam, J. J. (1995). Publication decisions revisited: The effect of the outcome of statistical tests on the decision to publish and vice versa. *American Statistician, 49*(1), 108-112.

# Peer review: Reviewer response for version 1

**DOI:** 10.21956/wellcomeopenres.16993.r36874

**Published:** 2019-11-12

**Authors:** Guy Vingerhoets

**Affiliations:** 1 Ghent University, Ghent, Belgium

By comparing monozygotic and dizygotic twin pairs on measures of hand and language laterality the authors aim to evaluate the genetic influence of these asymmetries. Consistent with previous reports a modest heritability effect of slightly over .20 was found for handedness, but for language laterality twin-cotwin correlations were close to zero. The authors conclude that heritability of language lateralization is low at best.

 This is a well written and methodologically sound and detailed study investigating the genetic effect on hand and language laterality. In the introduction the authors explain the rationale for their approach and review the available data on genetic variation of laterality. I would suggest to slightly rearrange this section as after shortly introducing genetic variation on handedness and language, they move on to describe the genetics of structural asymmetries for two paragraphs, to return to behavioral laterality in the next paragraph. Maybe it is better to treat structural and functional asymmetry more separately, with the latter being more relevant to the present study than the former. In general, the findings of these studies reveal only moderate effects which seems in contrast with the claim in the first sentence of the abstract.

 I’m not an expert in heritability research, but the approach of the authors seems methodologically sound. The number of twins included is high, and a power analysis addresses the feasibility to detect an effect. The sample is clearly described, and laterality assessment of handedness and language are explained in detail. The only detail that puzzles me is the period of interest of the fTCD procedure. In one paragraph a start and stop cue indicating a 10s period is mentioned during which the child describes the 12s cartoon. In another paragraph the period of interest is defined as 4 to 14s after the cue to speak, but this method was abandoned for the ‘whole period of interest’. Would that be the 10s period between both cues then?

 Results are illustrated with distribution plots and scatterplots and the results of the AE model are presented in the classical and ordinal version.

 In the discussion, the authors mention the consistent finding regarding handedness (genetic factors account for about 20% of the variance), and quickly move on to interpret the close to zero correlation for twin-cotwin language laterality findings.

 They start by questioning the validity of the fTCD derived language laterality index and raise three arguments in favor of their measure: (1) split-half reliability is high, (2) fTCD laterality indices (LI) reflect the expected population bias, and (3) the low heritability is consistent with other reports on structural and functional lateralization. They further argue that their findings cannot dismiss the possibility that there is a small but real genetic effect on language lateralization and that the sample may be too small to detect it. In other words, while their data suggest no role for genetic factors on language laterality, the authors leave open the possibility of a small (the title says negligible, which I find a somewhat subjective term) effect. The argument used for this interpretation is grounded on (potentially insufficient) sample size rather than (potentially invalid) measurement. While this might be the case, I would argue that fTCD measurement has several drawbacks the authors may wish to consider. Although not perfect, the reliability of fTCD is overall reasonable (Stroobant & Vingerhoets, 2001 ^1^; Vingerhoets & Stroobant, 2002 ^2^). But reliability is not validity. fTCD measures blood flow velocity in the basal part of the middle cerebral arteries. This artery supplies blood to most of the lateral surface of the brain or roughly 80% of each hemisphere. As a result, fTCD- derived LI’s offer only crude estimations of asymmetry as they will pick up velocity changes due to co-activation of many other mental components that take place in the MCA territory and that may not be related to language. The child has just seen a 12s cartoon and now must describe it. Attention, memory, visual imagery, movement(?), receptive and productive language areas all come into play and most of these functions will be associated with activation of the lateral cortex. Besides not being very region-specific, fTCD does not allow for the use of control-tasks that can correct for task-unspecific activation. My point is that fTCD-derived LI’s of language tasks may be sufficient to (reliably) reflect the population bias of left hemisphere language dominance, but that they may not be sufficient to provide valid markers of lateralization strength. Therefore, its use as a binary variable may be more successful than when used as a continuous variable. Note the fTCD index plotted in Figure 1: the mean LI lies between .20 to .30 in favor of the left hemisphere. This is not very lateralized which may be due to the joint activation of other mental functions that dilute the asymmetry of the language component. Compare this mean LI with the fMRI-derived mean value of about .65 in favor of the left hemisphere using a sentence production task based on cartoon drawings (Mazoyer *et al*. 2004 ^3^). Although the fMRI LI’s were based on whole hemisphere data, the application of a control task was able to filter out much of the non-relevant general mental activation, a procedure not possible with fTCD. Finally, the fMRI results were obtained in adults, not in 6 to 12-year-old children whose language lateralization may be more variable, in particular when they have language or literacy difficulties.

 This consideration should not do short to the excellent work presented in this paper. It is simply not feasible to place all these children in an MRI scanner. The results presented provide important information on the effect of genetic factors on language laterality in children. I broadly agree with the conclusion that while we cannot completely dismiss the idea that genes have no role in language laterality, its effect is likely to be modest. In order for that message to come across, the word ‘negligible’ might need some fine tuning.

Is the work clearly and accurately presented and does it cite the current literature?

Yes

If applicable, is the statistical analysis and its interpretation appropriate?

Yes

Are all the source data underlying the results available to ensure full reproducibility?

Yes

Is the study design appropriate and is the work technically sound?

Yes

Are the conclusions drawn adequately supported by the results?

Partly

Are sufficient details of methods and analysis provided to allow replication by others?

Partly

Reviewer Expertise:

Neuropsychology.

I confirm that I have read this submission and believe that I have an appropriate level of expertise to confirm that it is of an acceptable scientific standard, however I have significant reservations, as outlined above.

## References

[1] : Test-retest reliability of functional transcranial Doppler ultrasonography. *Ultrasound in Medicine & Biology*.2001;27(4) : 10.1016/S0301-5629(00)00325-2 509-514 10.1016/S0301-5629(00)00325-2 11368863

[2] : Reliability and validity of day-to-day blood flow velocity reactivity in a single subject: an fTCD study. *Ultrasound in Medicine & Biology*.2002;28(2) : 10.1016/S0301-5629(01)00506-3 197-202 10.1016/S0301-5629(01)00506-3 11937282

[3] : Gaussian mixture modeling of hemispheric lateralization for language in a large sample of healthy individuals balanced for handedness. *PLoS One*.2014;9(6) : 10.1371/journal.pone.0101165 e101165 10.1371/journal.pone.0101165 24977417PMC4076312

# Peer review: Reviewer response for version 1

**DOI:** 10.21956/wellcomeopenres.16993.r36877

**Published:** 2019-11-07

**Authors:** Clyde Francks

**Affiliations:** 1 Language and Genetics Department, Max Planck Institute for Psycholinguistics, Nijmegen, The Netherlands; 2 Donders Institute for Brain, Cognition and Behaviour, Radboud University, Nijmegen, The Netherlands

Congratulations to the authors for another important study in this field.

 I broadly agree with the authors that measures of brain and behavioural asymmetry tend to have low heritabilities. However, it is not clear to me that the heritability of language laterality in this study is negligible or zero, as asserted by the authors. The authors use appropriate caution in many parts of the paper, but other parts seem too strong, such as the current title of the paper, or the abstract, which give the impression that this study found negligible heritability.

 The authors report a heritability estimate of zero for their measure of language laterality, based on twin analysis. However, the confidence interval for this estimate is not given. This is not a large study, and the power calculations suggest that the confidence interval must have a substantial range. The authors can only be confident that the heritability falls within the confidence interval, not that it is zero or negligible. How high might it go, for example are the data compatible with 10-20% heritability? This would not be negligible. Related to this, the authors mention that a model with no genetic term gave as good a fit as one including it. Please include the goodness-of-fit statistics.

 I would like to make the authors aware of some relevant literature, some of which is from my own group (I may have missed opportunities to make them aware of these studies in recent years). There are two family- and/or twin-based analyses of the heritability of brain structural laterality, that were based on larger sample sizes than those cited by the authors.

 Kong X *et al[*ref-1]

 Guadalupe *et al* ^2^

 These studies show significant heritabilites up to 27% for various aspects of brain structural asymmetry, and indicate that gene mapping for these asymmetries may be fruitful.

 As regards handedness, there are two recent genome-wide association scan (GWAS) studies of handedness based on the UK biobank data of more than 330,000 subjects.

 kovel *et al* ^3^

 Wiberg *et al* ^4^

 These studies are over two orders of magnitude larger than the GWAS cited by the authors, and have identified significant genetic associations with left-handedness, offering some glimpses into the biology of the trait. Note that the SNP-based heritability of left-handedness was only around 2% in the UK Biobank data, but still significant due to the large sample size. This shows that a heritability even as low as 2% can be a basis for successful gene mapping, to deliver insights into trait biology, which is a point that the authors could acknowledge to give a balanced impression to the field. If the author’s data are compatible with even 5-10% heritability for language laterality (in terms of the confidence interval around their heritability estimate), then future gene mapping is certainly possible, that might help to reveal genetic-developmental mechanisms of laterality formation. My concern is that a title and abstract saying that heritability was negligible does not really capture this possibility helpfully for the field, nor the uncertainty within this study itself.

 Methods: I have no experience with data from functional transcranial Doppler sonography. I assume the authors have analyzed this in a correct and state-of-the-art way. However, Somers et al. (2015) ^5^ found a heritability of 31% for atypical language lateralisation (coded as a binary variable), using functional transcranial Doppler ultrasound in a multi-generational pedigree sample. If the same data processing pipeline from that study would be applied in the current study, might the results no longer be discrepant between the two studies?

 In general, measuring asymmetry necessarily involves calculating some kind of difference score, which means that error variance in both left and right measures can affect the asymmetry measure. This may partly explain the low heritabilities of asymmetry indexes, and the authors could acknowledge this. In brain structural analysis (of e.g. grey matter volumes, surface areas, thicknesses), we typically see higher heritabilities for L and R separately than for the asymmetry index (L-R)/(L+R). Might it be informative to calculate heritabilities for L and R, in the context of functional transcranial Doppler sonography? If L and R are not themselves heritable, there may be a problem with the approach.

 Abstract: The authors state that ‘it is widely assumed that individual differences in language lateralisation have a strong genetic basis’. Are there references to support this? I am not sure that many researchers working on the genetics of laterality have this impression (this one does not).

 The Discussion can be revised in parts, to reflect the recent literature indicated above.

 The authors wrote that ‘Our data are compatible with a more radical model in which language laterality is the consequence of a general population left-sided brain bias for language which does not show any individual variation.’ For clarity, it would help to make explicit that genetic variation is meant here. I agree that the author’s data are compatible with this model, but the limited sample size means that the data are also compatible with a heritability up to whatever level is encompassed by the confidence interval around the best estimate.

Is the work clearly and accurately presented and does it cite the current literature?

Partly

If applicable, is the statistical analysis and its interpretation appropriate?

Partly

Are all the source data underlying the results available to ensure full reproducibility?

Yes

Is the study design appropriate and is the work technically sound?

Yes

Are the conclusions drawn adequately supported by the results?

Partly

Are sufficient details of methods and analysis provided to allow replication by others?

Yes

Reviewer Expertise:

Human neurogenetics

I confirm that I have read this submission and believe that I have an appropriate level of expertise to confirm that it is of an acceptable scientific standard, however I have significant reservations, as outlined above.

## References

[1] : Mapping cortical brain asymmetry in 17,141 healthy individuals worldwide via the ENIGMA Consortium. *Proceedings of the National Academy of Sciences*.2018;115(22) : 10.1073/pnas.1718418115 E5154-E5163 10.1073/pnas.1718418115 PMC598449629764998

[2] : Human subcortical brain asymmetries in 15,847 people worldwide reveal effects of age and sex. *Brain Imaging Behav*.2017;11(5) : 10.1007/s11682-016-9629-z 1497-1514 10.1007/s11682-016-9629-z 27738994PMC5540813

[3] : The molecular genetics of hand preference revisited. *Sci Rep*.2019;9(1) : 10.1038/s41598-019-42515-0 5986 10.1038/s41598-019-42515-0 30980028PMC6461639

[4] : Handedness, language areas and neuropsychiatric diseases: insights from brain imaging and genetics. *Brain*.2019;142(10) : 10.1093/brain/awz257 2938-2947 10.1093/brain/awz257 31504236PMC6763735

[5] : A common polymorphism in the 5' UTR of ERCC5 creates an upstream ORF that confers resistance to platinum-based chemotherapy. *Genes Dev*.2015;29(18) : 10.1101/gad.261867.115 1891-6 10.1101/gad.261867.115 26338418PMC4579346
